# A Cas6-based RNA tracking platform functioning in a fluorescence-activation mode

**DOI:** 10.1093/nar/gkac014

**Published:** 2022-01-21

**Authors:** Feng Gao, Ke Zheng, You-Bo Li, Feng Jiang, Chun-Yu Han

**Affiliations:** Gene Editing Research Center, Hebei University of Science and Technology, Shijiazhuang, Hebei 050018, China; Gene Editing Research Center, Hebei University of Science and Technology, Shijiazhuang, Hebei 050018, China; School of Basic Medical Sciences, Hebei University, Baoding, Hebei 071000, China; Gene Editing Research Center, Hebei University of Science and Technology, Shijiazhuang, Hebei 050018, China; Gene Editing Research Center, Hebei University of Science and Technology, Shijiazhuang, Hebei 050018, China

## Abstract

Given the fact that the localization of RNAs is closely associated with their functions, techniques developed for tracking the distribution of RNAs in live cells have greatly advanced the study of RNA biology. Recently, innovative application of fluorescent protein-labelled Cas9 and Cas13 into live-cell RNA tracking further enriches the toolbox. However, the Cas9/Cas13 platform, as well as the widely-used MS2-MCP technique, failed to solve the problem of high background noise. It was recently reported that CRISPR/Cas6 would exhibit allosteric alteration after interacting with the Cas6 binding site (CBS) on RNAs. Here, we exploited this feature and designed a Cas6-based switch platform for detecting target RNAs in vivo. Conjugating split-Venus fragments to both ends of the endoribonuclease-mutated *Escherichia coli* Cas6(d*Ec*Cas6) allowed ligand (CBS)-activated split-Venus complementation. We name this platform as Cas6 based Fluorescence Complementation (Cas6FC). In living cells, Cas6FC could detect target RNAs with nearly free background noise. Moreover, as minimal as one copy of CBS (29nt) tagged in an RNA of interest was able to turn on Cas6FC fluorescence, which greatly reduced the odds of potential alteration of conformation and localization of target RNAs. Thus, we developed a new RNA tracking platform inherently with high sensitivity and specificity.

## INTRODUCTION

The current live-cell RNA tracking techniques fall into two types: fluorescence-enrichment (FE) type and fluorescence-activation (FA) type. An FE-type platform usually comprises two key components: a fluorescent protein-conjugated RNA binding protein (RBP-FP) that acts as a reporter or tracker, and a specific RBP binding sequence (RBS) genetically constructed on target RNAs. A prominent example of the FE-type is the MS2-MCP RNA tracking system in which MCP stands for MS2 phage coat protein (1). MCP binds a specific RNA stem-loop structure named MBS (MCP binding site). For tracking target RNAs in living cells, RNAs of interest are to be tagged with multiple copies of MBS for recruiting fluorescent protein-conjugated MCP (MCP-FP), thus making the target RNAs visible. The MS2-MCP system is the first invented and the most widely used RNA tracking system. After that, various analogous FE-type RNA tracking systems were established, including PP7-PCP, and λN (2,3). However, the FE-type platforms usually have a weakness of high background noise. For enhancing the signal-to-noise ratio (SNR), a rather high copy number (usually >24 copies, >1000 bp) of exogenous tandem-repeated RBSes are used to tag the target RNAs (1,2), which is complex in genetic manipulation and potentially raises the possibility of altering the structure and localization of the target RNAs.

Recently, Cas9 and Cas13 gene editing tools were proposed to RNA imaging (4–6). Benefiting from the guide RNA (gRNA)-mediated RNA targeting, FP-conjugated Cas9/Cas13 could track any RNA of interest. However, given the fact that at least 12 copies of MBS are required for MS2-MCP system to gain an acceptable SNR (1), application of quite a few gRNAs targeting different motifs of a single target RNA is conceivably required for a Cas9/Cas13 RNA imaging platform to achieve decent SNR. Besides, the relatively large size of Cas9/Cas13 is a concern, since accumulation of multiple Cas9/Cas13 reporters onto a single RNA would possibly compromise the integrity of the conformation and localization of the RNA of interest. So far, the MS2-MCP platform is still the best of FE-type RNA imaging tool.

All FE-type RNA tracking platforms inherently suffer from high background noise due to the application of florescent reporters. This concern is partly addressed by the FA-type RNA tracking/imaging platforms. The FA-type platforms are based on bimolecular fluorescence complementation (BiFC). For example, MCP and PCP (an RNA binding protein analogous to MCP) were fused with N- and C- fragment of a certain FP, respectively; the resultant two reporters, MCP-FPN and PCP-FPC, would not reconstitute a fully functional florescent protein until they are brought together by adjacent MBS and PBS (PCP binding site) on an RNA target (7,8). As such, the FA-type platforms have much improved SNR. Undoubtedly, the current FA-type platforms, such as BiFC or TriFC RNA tracking systems (8), outstrip any FE-type tools in sensitivity. However, in contrast to the 12 × MBS tag which MS2-MCP system requires, even larger RNA tag, e.g. 12 × MBS-PBS (1400 nt), is frequently used for the current FA-type platforms (7,8). Insertion of this large exogenous RNA sequence is liable to cause problems mentioned above.

Cas6 is a core component of type I-E CRISPR complex. It binds and cleaves pre-crRNA by recognizing a specific stem-loop RNA element designated as CBS (Cas6 binding site) (9,10). CBS-binding induces an allosteric change of Cas6, leading to juxtaposing its N and C termini (9). Employing this feature, we engineered an *Escherichia coli-*originated Cas6 (*Ec*Cas6) protein into an FA-type reporter. The catalytic domain-mutated *Ec*Cas6 (d*Ec*Cas6) was appended at its N- and C-termini with the non-fluorescent moieties of protein Venus, Venus-N (VN) and Venus-C (VC), respectively. The resultant chimera VN-d*Ec*Cas6-VC was not fluorescent until binding to CBS on the RNAs of interest. Because Fluorescence Complementation is mediated by the allosteric switch of Cas6, we name this platform as Cas6 based Fluorescence Complementation (Cas6FC). Thus, we designed a new FA-type RNA tracking platform with favorable sensitivity and specificity.

## MATERIALS AND METHODS

### Plasmids

The annotations and important sequences of all the plasmids used in this study are presented in Supplementary Figure S1–S18.

### Cell culture and transfection

HEK293T, COS-7 and HeLa cell lines were purchased from China Center for Type Culture Collection (CCTCC). HEK293T and HeLa cells were cultured in Dulbecco's modified Eagle's medium (Gibco) supplemented with 10% fetal bovine serum (Gibco). COS-7 cells were cultured in RPMI Medium 1640 (Gibco) supplemented with 10% fetal bovine serum (Gibco). Lipofectamine^®^ 2000 Reagent (Invitrogen) was used for COS-7 cell transfection according to the manufacturer's instruction with the modified dose of 1 μl per well on 24-well plate. Entranster™-R4000 (Engreen Biosystem) was used for HEK293T and Hela cell transfection according to the manufacturer's instruction with the modified dose of 0.5 μl per well on 24-well plate. The plasmid usage was listed in Table 1.

**Table 1.** Plasmid usage for transfection

Assay	Plasmid dosage and purpose	Relative to figure
Turn-off reporter system for confirming d*Ec*Cas6 inactivation in HEK293T cells	100 ng CBS-EGFP-N1 + 300 ng pDsRed-Monomer-C1 as negative control	Figure 1A
	100 ng CBS-EGFP-N1 + 300 ng *Ec*Cas6-GK	
	100 ng CBS-EGFP-N1 + 300 ng d*Ec*Cas6-GK	
Turn-on reporter system for confirming d*Ec*Cas6 inactivation in HEK293T cells	100 ng Rm-16 × CBS-Lin28-C1 + 300 ng EGFP-C1 as negative control	Figure 1B
	100 ng Rm-16 × CBS-Lin28-C1 + 300 ng *Ec*Cas6-GK	
	100 ng Rm-16 × CBS-Lin28-C1 + 300 ng d*Ec*Cas6-GK	
d*Ec*Cas6-EGFP mediated *DsRed-Monomer-20 × CBS* mRNA imaging in HEK293T cells	50 ng *Ec*Cas6-EGFP-N1 + 600 ng pDsRed-Monomer-C1 or Rm-20 × CBS-C1	Figure 1C
	50 ng dCas6-EGFP-N1 + 600 ng pDsRed-Monomer-C1 or Rm-20 × CBS-C1	
Cas6FC mediated *DsRed-Monomer-20 × CBS* mRNA imaging in HEK293T cells	100 ng VN-d*Ec*Cas6-VC-GK + 400 ng pDsRed-Monomer-C1 or Rm-20 × CBS-C1	Figure 2B
Correlation of smiFISH and Cas6FC for *ACTB-16 × CBS* mRNA imaging in HEK293T cells	200 ng VN-d*Ec*Cas6-VC-GK + 800 ng Actin-16 × CBS-GK	Figure 2D
Correlation of FISH and VN- Cas6FC for *hTERC-16 × CBS* lncRNA imaging in Hela cells	200 ng VN-d*Ec*Cas6-VC-GK + 800 ng hTERC-16 × CBS-GK	Figure 2E
Validation of Cas6FC background-free property in HEK293T cells using flow cytometry	50 ng Actin-GK + 400 ng pDsRed-Monomer-C1 as ‘red channel positive control’ for fluorescent compensation and as ‘control group’ for self-fluorescence	Figure 3A
	50 ng VN-d*Ec*Cas6-VC-GK + 400 ng Actin-16 × CBS-GK as ‘green channel positive control’ for fluorescent compensation	
	50 ng VN-d*Ec*Cas6-VC-GK + 400 ng pDsRed-Monomer-C1 as ‘without CBS group’ for VN-d*Ec*Cas6-VC background fluorescence	
	50 ng VN-d*Ec*Cas6-VC-GK + 400 ng Rm-20 × CBS-C1 as ‘with CBS group’ for VN-d*Ec*Cas6-VC specific fluorescence	
Verifying the minimum required copy number of CBS for Cas6FC mediated RNA imaging in HEK293T cells	100 ng VN-d*Ec*Cas6-VC-GK + 200 ng U6-1 × CBS	Figure 3B
Sensitivity of Cas6FC system for tracking CMV promoter-driven target RNAs.	VN-d*Ec*Cas6-VC-GK + 400 ng Actin-GK or Actin-n × CBS-GK series plasmids (n = 1,2,4,8,16).	Figure 3C
Investigating specificity of CBS-induced Cas6FC fluorescence	100 ng VN-d*Ec*Cas6-VC-GK + 400 ng Rm-4 × CBS variants-C1	Figure 4

Note: In the process of naming plasmid, ‘DsRed-Monomer’ is abbreviated as ‘Rm’, e.g. Rm-20 × CBS-C1.

### Fluorescence *in situ* Hybridization of RNA

Hela cells and HEK293T cells were cultured in 24-well plates with poly-d-lysine coverslips, and then these cells were co-transfected with 200 ng VN-d*Ec*Cas6-VC-GK and 800 ng Actin-16 × CBS-GK or hTERC-16 × CBS-GK. Single molecule inexpensive fluorescence *in situ* hybridization (smiFISH) was employed for RNA imaging (11). The sequences of primary probes and FLAP probes were listed in Supplementary Table S1. The smiFISH was performed according to the previously reported method. Briefly, cells were fixed with 4% PFA for 15 min at 37°C and washed with 1 × PBS (pH7.4) for 5 min three times, followed by permeabilization with 0.5% Triton X-100 for 15 min, and these cells were rewashed with 1 × PBS (pH7.4) for 5 min three times. Cells were incubated in hybridization solution at 37°C for 12 h. After hybridization, the samples were washed with 15% formamide (in 1 × SSC) at 37°C for 30 min three times, stained with 0.1 μg/ml DAPI at room temperature for 10 min, and mounted in antifading mounting medium (Solarbio).

### Flow cytometry assay

The HEK293T cells in 24-well glass-bottom plate were transfected with plasmids according to the experiment design (Table 1). Four replications were performed for each experiment sample, of which two replicates were observed using confocal microscope, the other two replicates were subjected to flow cytometry analysis. At 24 h post transfection, the HEK293T cells were digested with 0.25% (w/v) trypsin (in 1 × PBS pH7.4) and analyzed with BD FACSCelesta flow cytometer. Prior to analyzing background and CBS-induced specific fluorescence of VN-d*Ec*Cas6-VC, the fluorescent compensation was performed with Actin-GK + pDsRed-Monomer-C1 transfected cells used for red fluorescence channel compensation, and VN-d*Ec*Cas6-VC-GK + Actin-16 × CBS-GK transfected cells used for green fluorescence channel compensation, respectively. The parameters for flow cytometry analysis were as follows: 440 V laser voltage for red fluorescence channel (PE-Texas Red) and 320 V laser voltage for green fluorescence channel (FITC), respectively. Fluorescence compensation parameters were 4.8 for red fluorescence channel and 12.6 for green fluorescence channel, respectively. A total of 10 000 single living cells were sorted from all the cells according to FSC (Forward Scatter) and SSC (Side Scatter). As the co-transfected cells should express DsRed-Monomer fluorescent protein, the red fluorescence positive (Red^+^) cells were then sorted and taken for analysis of VN-d*Ec*Cas6-VC background and specific fluorescence. The Actin-GK + pDsRed-Monomer-C1 co-transfection group worked as control group; the VN-d*Ec*Cas6-VC-GK + pDsRed-Monomer-C1 co-transfection group (named as without CBS group) was used for analyzing initial background fluorescence; the VN-d*Ec*Cas6-VC-GK + Rm-20 × CBS-C1 group (named as with CBS group) was used for analyzing specific fluorescence. The maximum fluorescence intensity of Red^+^ control group cells was used as a threshold for determining initial background fluorescence or specific fluorescence. The Red^+^ without CBS group cells fluorescence whose intensity was higher than the above threshold was determined as initial background fluorescence; the Red^+^ with CBS group cells fluorescence whose intensity was higher than the above threshold was determined as specific fluorescence.

### RNA imaging

For RNA imaging in living cells, the transfected cells in 24-well plates were observed on ZEISS Vert.A1 microscope platform at 24 h post transfection. For FISH imaging, the coverslips were mounted and observed on ZEISS Vert.A1 microscope platform. Exposure time of each fluorescence channel was set as 5 s, and that of bright field as 100 ms. For the observation of fixed cells by using confocal microscope, the transfected cells were fixed with 4% PFA for 15 min at 37°C, and washed with 1 × PBS (pH7.4) for 5 min three times, followed by permeabilization with 0.5% Triton X-100 for 15 min, and these cells were rewashed with 1 × PBS (pH 7.4) for 5 min three times. After being stained with 0.1 μg/ml DAPI and washed with 1 × PBS (pH7.4) for 5 min three times, the cells were observed with Nikon A1R HD25 Confocal Microscope.

## RESULTS

### *Escherichia coli (Ec)* Cas6 could be used for *in vivo* RNA tracking

**Figure 1.**
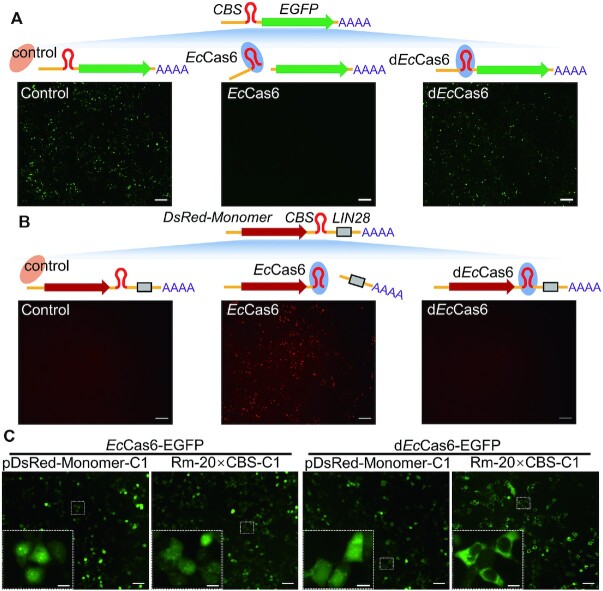
*Escherichia coli* Cas6 (*Ec*Cas6) could be utilized for eukaryotic RNA imaging. (**A**, **B**) Verification of CBS binding activity and catalytic activity of *Ec*Cas6 and d*Ec*Cas6 using a (A) ‘turn-off’ *CBS-EGFP* reporter system or a (B) ‘turn-on’ *DsRed-Monomer-CBS* reporter system in HEK293T cells. A separate RFP/EGFP-expressing vector which did not contain either the LIN28 or the CBS sequence was co-transfected as an internal control. Scale bar, 200 μm. (**C**) HEK293T cells co-transfected with a vector expressing *DsRed-Monomer* mRNA appended with (Rm-20 × CBS-C1) or without (pDsRed-Monomer-C1) CBS sequence, and a plasmid expressing either *Ec*Cas6-EGFP or d*Ec*Cas6-EGFP, were imaged for *DsRed-Monomer* mRNA intracellular distribution. Scale bar for the low power images, 50 μm; scale bar for the high power images, 10 μm. The dosages of plasmids used were listed in Table 1. Representative pictures from 3 times of independent experiments.

Due to their CBS binding capability, members of Cas6 family have the potential to be used as an FE-type RNA tracking tool. To test this possibility, we selected Cas6 derived from *Escherichia coli* (*Ec*Cas6) and investigated its application in tracking RNAs in mammalian cells at physiological temperature (37°C). We constructed a catalytically inactive *Ec*Cas6 mutant (an H→A mutation at Position 20 according to a previous study (12), and designated it as d*Ec*Cas6 (dead *Ec*Cas6). To confirm that a. *Ec*Cas6 could recognize and cleave CBS and b. d*Ec*Cas6 lost the endoribonuclease activity but retained its binding activity to CBS in mammalian cells, we test them in *de novo* designed turn-off and turn-on reporter systems. In the turn-off system, 1 × CBS sequence was inserted into the 5′-UTR of the *EGFP* mRNA. Since the 5′ cap is pivotal for mRNA translation and *Ec*Cas6-mediated cleavage would remove 5′-cap from *EGFP* mRNA, a successful *Ec*Cas6-CBS interaction would lead to reduction of EGFP fluorescence intensity. In HEK293T cells, co-transfection of *Ec*Cas6-expressing vector significantly reduced EGFP fluorescence intensity, which was not observed when d*Ec*Cas6-expressing construct was used instead (Figure 1A). In the turn-on system, a sequence of 16 × CBS and a sequence of 2 × LIN28 RNA nuclear retaining signal were sequentially inserted into the 3′-UTR of the *DsRed-Monomer* (abbreviated as *Rm*) mRNA. LIN28 RNA nuclear retaining signal would retain the host mRNA in nucleus (13); as a consequence, the DsRed-Monomer fluorescent protein could not be translated. Removal of LIN28 RNA nuclear retaining signal by *Ec*Cas6 cleavage could release the *DsRed-Monomer* mRNA for translation in the cytoplasm. Indeed, transfection of the Rm-CBS-LIN28-C1 vector showed nearly undetectable DsRed-Monomer protein expression when evaluated by a fluorescent microscope. However, introduction of *Ec*Cas6 significantly enhanced the fluorescence of DsRed-Monomer protein, reflecting a successful *Ec*Cas6-mediated cleavage. As predicted, introduction of d*Ec*Cas6 to the cells could not turn on the expression of DsRed-Monomer protein (Figure 1B). Taken together, we verified effectiveness of the *Ec*Cas6-CBS system in mammalian cells and the d*Ec*Cas6 did lose enzymatic activity.

The mutation on d*Ec*Cas6 should only abrogate its endoribonuclease activity and will keep the CBS-binding ability. Employing this property of d*Ec*Cas6, we genetically labeled d*Ec*Cas6 with EGFP and used this fluorescent chimera protein to track target RNAs which were genetically tagged with CBS. As shown in Figure 1C, without the 20 × CBS tag on the target *DsRed-Monomer* mRNA, both *Ec*Cas6-EGFP and d*Ec*Cas6-EGFP could be visualized in both cytoplasm and nucleus. With the 20 × CBS tag hanged on the target mRNA, only the cells co-transfected with the d*Ec*Cas6-EGFP but not the *Ec*Cas6-EGFP construct could localize the target mRNA in the cytoplasm. Thus, d*Ec*Cas6, when labeled with a fluorescent protein, could be applied for RNA detection as an FE type tool.

### Establishment of a VN-d*Ec*Cas6-VC based FA-type RNA tracking platform

Similar to other FE-type RNA tracking platforms, the d*Ec*Cas6-EGFP technique also suffers from a high-background problem with unfavorable signal-to-noise ratios. It was reported that binding to CBS induced a conformational change of *Thermus thermophilus* Cas6 (*Tt*Cas6), resulting in juxtaposing the N and C termini of *Tt*Cas6 (9) (Supplementary Figure S19). This finding prompted us to investigate the possibility that Cas6 could be exploited as an FA platform for tracking RNA *in vivo*. However, the premium working temperature for *Tt*Cas6 is around 65°C, precluding its usage in mammalian cells. *E. coli* propagate at 37°C. Based on the similarity of the structures between *Ec*Cas6 and *Tt*Cas6 (Supplementary Figure S19B) (14), we postulated that *Ec*Cas6 could be exploited for this purpose in mammalian cells.

To construct an FA type fluorogenic reporter, we linked split-FP fragments to the ends of d*Ec*Cas6 protein with diverse peptide linkers, generating a series of d*Ec*Cas6-split FP fusion proteins (Supplementary Figure S20–S22). The FA type fluorogenic reporter should be non-fluorescent at free state but would turn fluorescent upon binding target RNAs. According to this criterion, a desired fusion protein, termed as VN-d*Ec*Cas6-VC (53 kDa), was isolated.

**Figure 2.**
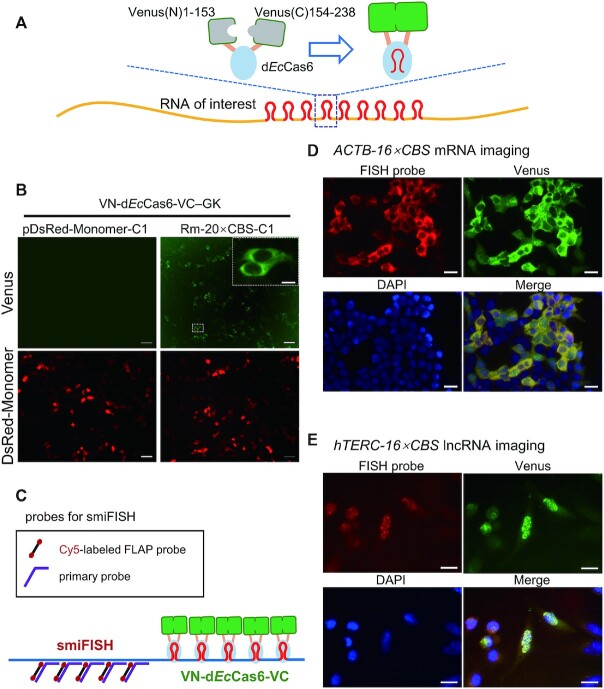
VN-d*Ec*Cas6-VC could be utilized for FA-mode RNA imaging. (**A**) Graphical illustration of conjectural mechanism of RNA tracking by a Cas6FC platform. (**B**) Visualizing the *DsRed-Monomer-20 × CBS* mRNA with the Cas6FC in HEK293T cells. Scale bar for the low power images, 50 μm; scale bar for the high power image, 10 μm. (**C**) Graphical illustration of the strategy to verify Cas6FC-mediated RNA tracking by FISH. (**D**) Correlation of *ACTB* mRNA tracking signals derived from Cas6FC and FISH in HEK293T cells. Scale bar, 20 μm. (**E**) Correlation of *hTERC* lncRNA tracking signals derived from Cas6FC and FISH in HeLa cells. Scale bar, 20 μm. The dosages of plasmids used were listed in Table 1. Representative pictures from 3 times of independent experiments.

This VN-d*Ec*Cas6-VC fusion protein consists of sequentially linked Venus-N terminal fragment (1–153), linker1, d*Ec*Cas6, linker2 and Venus-C fragment (154–238) (Figure 2A and Supplementary Figure S7). To demonstrate the proof of concept, we transfected HEK293T cells with a plasmid expressing VN-d*Ec*Cas6-VC; together, a vector expressing either the natural *DsRed-Monomer* mRNA or a CBS-tagged *DsRed-Monomer* mRNA was co-transfected. The 20 × CBS tag was appended at the 3′ terminal of the mRNA to minimize its influence on RNA conformation and distribution. VN-d*Ec*Cas6-VC did not display self-fluorescence when untagged *DsRed-Monomer* mRNA was transcribed (Figure 2B). However, the signal of fluorescent Venus could be successfully detected in the cytoplasm when CBS-tagged *DsRed-Monomer* mRNA was used. Appending the 20 × CBS tag on the 3′ terminal did not affect translation of the mRNA since comparable DsRed-Monomer protein signals could be observed between the two groups (Figure 2B). Similar results were obtained when COS-7 and Hela cell lines were tested (Supplementary Figure S23), indicative of the applicability of the d*Ec*Cas6 based Fluorescence Complementation RNA-tracking platform to a broad range of mammalian cells. Different from former BiFC or TriFC FA type RNA tracking platform, whose Fluorescence complementation is mediated by indirect protein-protein interactions through juxtaposed binding with their RBSes; VN-d*Ec*Cas6-VC is a one protein, RBS triggered, allosteric switch-based FC (Fluorescence complementation) RNA tracking platform. As all its unique characters are empowered by the Cas6 protein, we name this new RNA tracking platform as Cas6 based Fluorescence Complementation (Cas6FC).

Finally, to validate the authenticity of Cas6FC RNA tracking, we made a side-by-side comparison between the Cas6FC platform and RNA smiFISH (single molecule inexpensive fluorescence *in situ* hybridization) technique. To exclude the potential bias introduced by technique discrepancy between them, the FISH probes were designed aiming at the natural non-CBS sequence of the target RNAs and the same batch of cells were tested by both Cas6FC and RNA smiFISH (Figure 2C). In this case, the FISH probes were linked with Cy5, a fluorophore with emission wavelength distant from Venus, enabling co-localization analysis. The *ACTB* mRNA of HEK293T cells and the *hTERC* (human telomerase RNA component) lncRNA of Hela cells were chosen as the target RNAs for the comparison. The mRNA of *ACTB* is cytoplasm-localized and encodes a housekeeping cytoskeleton protein (15) and the nucleus-localized *hTERC* RNA is associated with cancer cell proliferation (16). The labeling patterns of both methods were examined by confocal microscopy. The highly consistent rendering between Cas6FC and RNA smiFISH in tracking RNAs at both targets (Figure 2D and E) warrants the usage of the Cas6FC platform in probing RNAs in mammalian cells. Also, these results demonstrated that the strategy of tagging CBS on the 3′ terminal would not affect natural localization of the targeted RNAs.

### Cas6FC is a sensitive RNA tracking system

**Figure 3.**
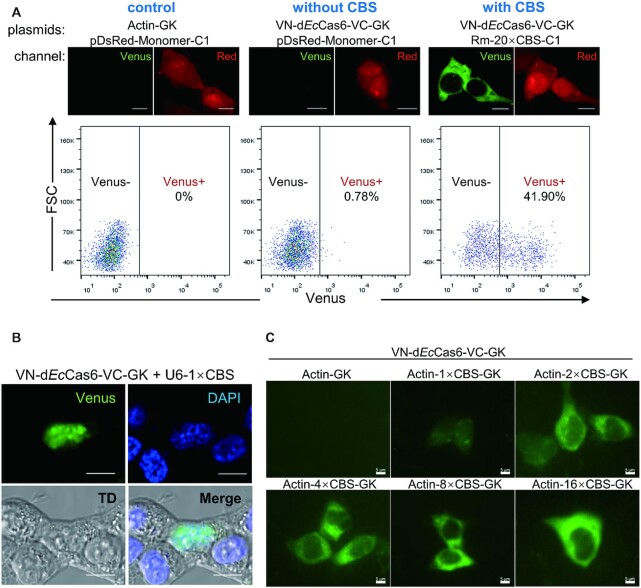
The sensitivity of the Cas6FC platform. (**A**) HEK293T cells were transfected with vectors expressing either pDsRed-Monomer-C1 or Rm-20 × CBS-C1. Cells were also transfected with the plasmids expressing β-actin (as a control) or VN-d*Ec*Cas6-VC. Twenty-four hours later, DsRed-Monomer and Venus expression was analyzed by confocal microscopy (Upper). In parallel, Red^+^ cells were gated for analyzing Venus signal by flow cytometry. Representative pictures from 3 independent experiments were shown. (**B**) A 29nt-long RNA carrying only one copy of CBS was transcribed under the RNA polymerase III promoter in HEK293T cells. The Cas6FC system could localize it in the nucleus. Scale bar, 10 μm. (**C**) *ACTB* mRNAs carrying 0×, 1×, 2×, 4×, 8× or 16× CBS was transcribed under an RNA polymerase II promoter in HEK293T cells. The sensitivity of the Cas6FC system to CBS number was examined by fluorescence microscopy. Scale bar, 5 μm. The dosages of plasmids used were listed in Table 1. Representative pictures from three independent experiments were shown.

To further evaluate the sensitivity of the Cas6FC platform in tracking target RNAs, we utilized flow cytometry which is relatively more sensitive in differentiating signal from noise than fluorescent microscopy. Compared to the background reference provided by the cells co-transfected with Actin-GK vector (expressing β-actin protein as control) and pDsRed-Monomer-C1 vector (expressing *DsRed-Monomer* mRNA and protein), the cells co-transfected with the VN-d*Ec*Cas6-VC-GK vector (expressing the reporter of Cas6FC platform) and pDsRed-Monomer-C1 vector had almost no detectable Venus signal (0.78% positive cells versus 0% of background) (Figure 3A). In contrast, 41.9% cells in the group co-transfected with the VN-d*Ec*Cas6-VC-GK vector and Rm-20 × CBS-C1 vector (expressing *DsRed-Monomer-20 × CBS* mRNA and DsRed-Monomer protein) displayed strong Venus fluorescence. Thus, the VN-d*Ec*Cas6-VC made negligible noise in the absence of CBS sequence in target RNAs.

Simply increasing the copy number of RBP binding motif could improve the sensitivity of FE type RNA tracking platforms. For instance, 24 × MBS (MCP binding sites) were often used in the MS2-MCP system to achieve optimal signals (1). However, a concern that inserting multiple copies of RBS to target RNAs would alter the structure and/or distribution of RNAs always haunts. We were thus set to determine the minimal RBS (CBS in this case) copies required for the Cas6FC platform. To this end, we first tested the sensitivity of Cas6FC in detecting a 29nt-long RNA carrying only one copy of CBS. This short RNA was transcribed under human U6 small nuclear RNA promoter (RNA polymerase III promoter) which is superior in generating short transcripts with high output. To our surprise, the 1 × CBS RNA could be conspicuously beheld in HEK293T cells (Figure 3B). The signal was exclusively located to the nucleus, in line with the fact that U6 promoter-driven RNAs lack the 5′ cap and Poly A tail of mRNAs and preferentially distribute in nucleus. Next, we examined the dose effect of CBS on detecting target mRNA driven by a CMV promoter (RNA polymerase II promoter) (Figure 3C). The intensity of fluorescence increased in a CBS copy number-dependent manner, and again, the mRNA carrying one copy of CBS was visible in the Cas6FC platform. In conclusion, as minimal as 1 × CBS (∼29nt) could grant detectability of the Cas6FC platform. Insertion of such a short tag could potentially simplify genetic manipulation and at the same time significantly minimize the possibility of interfering with the folding and distribution of target RNAs.

### The specificity of the Cas6FC RNA tracking platform

**Figure 4.**
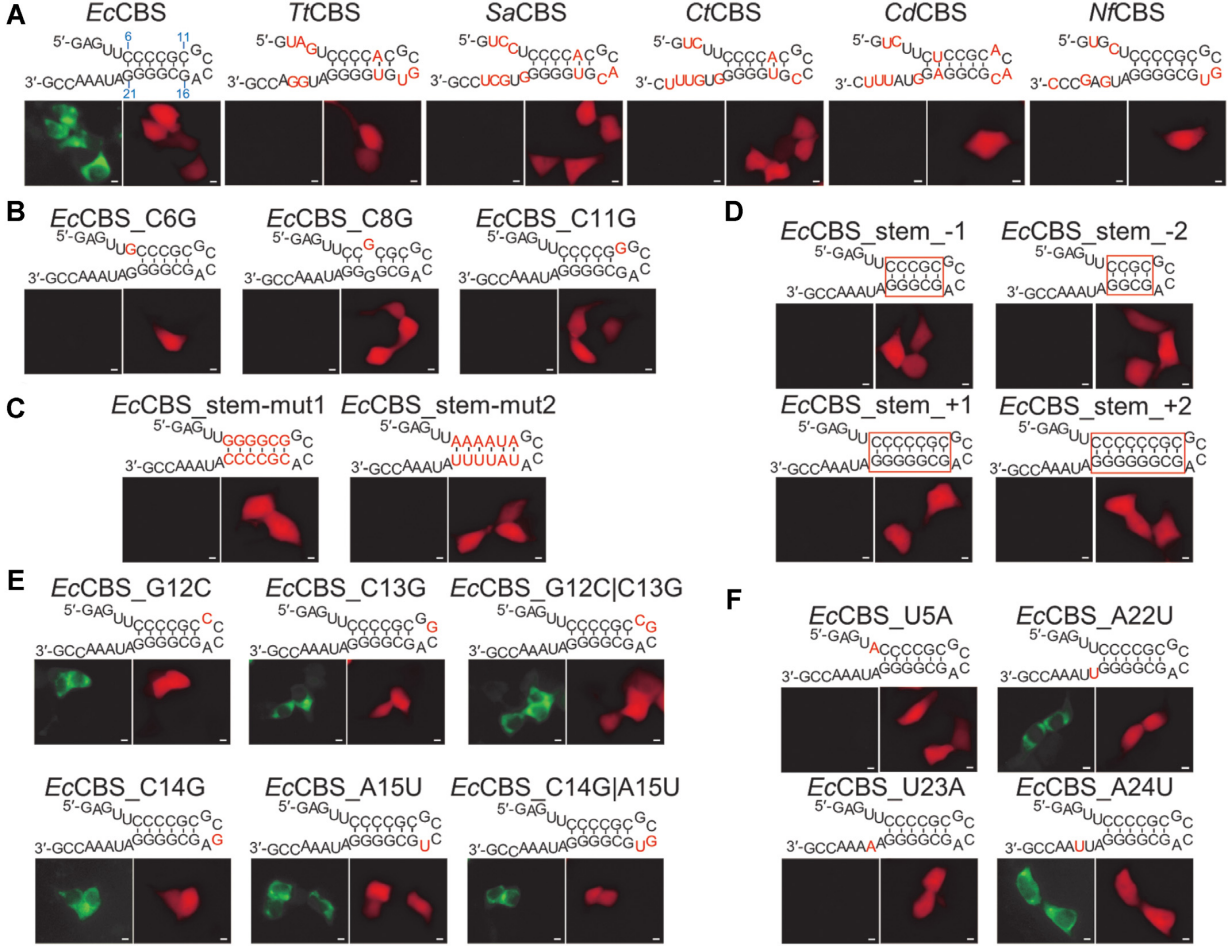
The specificity of the Cas6FC platform. (**A**) Interactions between VN-d*Ec*Cas6-VC and *Ec*Cas6 cognate binding sites (CBSes) were examined in live cells. The nucleotides different from *Ec*CBS on the cognate CBSes were highlighted in red. *Tt* for *Thermus thermophilus; Sa* for *Salinispora Arenicola; Ct* for *Chlorobium tepidum; Cd* for *Corynebacterium diphtheriae. Nf* for *Nocardia farcinica*. (**B**) The influences of stem-bulge mutations in *Ec*CBS on VN-d*Ec*Cas6-VC recognition were examined. (**C**) The influences of transition and transversion mutations of *Ec*CBS’s stem-localized nucleotides on VN-d*Ec*Cas6-VC recognition were examined. (**D**) The influences of stem length of *Ec*CBS on VN-d*Ec*Cas6-VC recognition were examined. (**E**) The influences of loop-localized nucleotides mutations in *Ec*CBS on VN-d*Ec*Cas6-VC recognition were examined. (**F**) The influences of non-stem-loop interacting nucleotide mutations in *Ec*CBS on VN-d*Ec*Cas6-VC recognition were examined. The mutated nucleotides were highlighted in red. Scale bar, 5 μm. Representative pictures from three independent experiments were shown. The dosages of plasmids used were listed in Table 1.

A successful application of the Cas6FC RNA tracking system partly depends on its specificity to the signature CBS binding sequence. To understand the species specificity of CBSes to *Ec*Cas6, we first analyzed the interactions between VN-d*Ec*Cas6-VC and CBS cognates *in vivo*. Five CBSes derived from different species were selected for this purpose (their nucleotide sequences and the corresponding Cas6 amino acid sequences were shown in Supplementary Table S2). Compared to *Ec*CBS, these CBSes differed in 7–11 nucleotides (Figure 4A). Four copies of each cognate CBS, including *Ec*CBS itself, were individually appended into the 3′UTR of the *DsRed-Monomer* gene. The results showed that the d*Ec*Cas6 based FC platform was highly faithful to *Ec*CBS since none of the CBS derived from other species could induce Venus signal (Figure 4A).

A previous work revealed that the CBS RNA motif had a hairpin conformation and its stem was critical for Cas6 recognition (17), different from the widely used MBS (for MS2) and PBS (for PCP) RNA tracking platforms which more relied on the specificity of the nucleotide sequences on the loop structure (18,19). We next studied the sequence specificity of *Ec*CBS for VN-d*Ec*Cas6-VC interaction. Based on their positions on the hairpin structure of *Ec*CBS, the nucleotides were divided into three groups: stem-localized nucleotides (Positions 6–11 and 16–21), loop-localized nucleotides (Positions 12–15) and the rest non-stem–loop positions. Accordingly, a series of *DsRed-Monomer-4 × mutant Ec*CBS gene embedded vectors were constructed. To test the specificity of the stem-localized nucleotides, we individually introduced single-nucleotide bulge mutations, transition/transversion mutations and stem-length mutations. As shown in Figure 4B, all the single-nucleotide bulge mutations, no matter occurring at the ends (C6G or C11G) or the middle (C8G) of the stem, blocked their interaction with VN-d*Ec*Cas6-VC, manifested by lack of fluorescence. Similarly, either transition/transversion mutation or stem-length mutation could completely abrogate their VN-d*Ec*Cas6-VC interaction (Figure 4C and D), even though these *Ec*CBS mutants retained the stem-loop structure. These mutagenesis assays substantiate the importance of the identity of nucleotides composing the stem structure in Cas6 recognition, in agreement with a structure-based projection predicting that the phosphate backbones of the nucleotides at positions 16–21 on *Ec*CBS interact with d*Ec*Cas6 through both electrostatic force and hydrogen bonds (Supplementary Figure S24A).

In contrast to the stem nucleotides, mutation of any single nucleotide residing in the loop structure had minimal effects on its interaction with VN-d*Ec*Cas6-VC (Figure 4E). According to the structure-based projection, both C14 and A15 putatively bind to d*Ec*Cas6 with hydrogen bonds (Supplementary Figure S24B). However, a C14G|A15U double mutant did not affect its interaction with VN-d*Ec*Cas6-VC, either (Figure 4E). These data indicate that nucleotides in Position 12–15 are trivial in interacting with VN-d*Ec*Cas6-VC (Figure 4E).

As for the third group of nucleotides, U5, A22, U23 and A24 were predicted to interact with *Ec*Cas6 with hydrogen bonds (Supplementary Figure S24B). However, A22 should not be crucial for interaction since it is supposed to bind to His20 of *Ec*Cas6, a homolog of His26 of *Tt*Cas6 (Supplementary Figure S24C and D); however, we constructed d*Ec*Cas6 by replacing His20 to Ala20 and d*Ec*Cas6 was still successfully used for probing CBS-carrying RNA (Figure 1C). Indeed, A22U mutation was not found to impede the interaction (Figure 4F). Similarly, A24U mutation had no effect, either. However, mutation at either U5 (to A) or U23 (to A) abrogated the development of Venus signal. Thus, the nucleotides at Positions 5 and 23, together with those stem-localized nucleotides, of *Ec*CBS appear irreplaceable for the d*Ec*Cas6 based RNA tracking platform (Cas6FC).

Finally, to interrogate potential off-target effect resulting from CBS-like sequence in mammalian transcriptomes, we analyzed occurrence frequencies of *Ec*CBS core motif in the transcriptomes of several mammalian species. According to our data above and a previous study (14), the core motif of *Ec*CBS for interacting with VN-d*Ec*Cas6-VC is between Position 4–24, i.e. 5′-UUCCCC GCNNN NGCGG GGNUN-3′. This core motif was aligned to RefSeq_RNA databases (NCBI) of *Homo sapiens*, *Rattus norvegicus* and *Mus Musculus*. It turned out that no endogenous RNAs derived from these three mammalian species carry this core *Ec*CBS motif. Taken together, the Cas6FC platform has advantages in both sensitivity and specificity over the current mainstream FE and FA RNA tracking platforms.

## DISCUSSION

By labeling an RBP with an FP, RBS-tagged RNA targets can be detected. Such a tracking system is simple but unavoidably introduces high background noise which is always a weakness of the FE-based platforms. In contrast, fluorescence complementation (FC) technique could solve this problem with much improved signal-to-noise ratio. As such, FA type platforms, including BiFC and TriFC RNA tracking systems, have achieved prominent progress. However, for achieving an ideal performance in using the current FA platforms, a large tag carrying multiple RBS repeats has to be inserted in the target RNAs, which could potentially alter the structure or location of the target RNAs.

As a prototype of fluorescence complementation (FC), protein BiFC makes use of protein-protein interaction to reconstruct fluorescence; current BiFC or TriFC RNA tracking platforms adopted this method, relying on juxtaposing two RBPs which carries the moieties of one fluorescent protein by binding to the adjacent corresponding RBSes. It thus imposes a requirement of optimal distance between the RBSes on the target RNAs: if designed too far, the fluorescent protein could not be successfully assembled; if too close, docking of two RBPs will be limited by room. Current solution is to insert multiple repeats of RBSes, aiming at providing more opportunity for the occurrence of RBP interaction. However, increasing the size of inserts is liable to disrupting the genuine structures of RBS as well as target RNAs which may in addition abrogate RBP binding. In this study, we developed an *Ec*Cas6-based FA-type RNA tracking platform (referred to as Cas6FC) to overcome these problems. The Cas6FC platform reconstructs a split fluorescence by employing the feature of allosteric alteration after Cas6-RBS binding. In this case, one RBP and one RBS can serve as a fully functional unit of FC, avoiding the need of designing two distinctive RBSes on one target RNA. Our data indicate that as minimal as one RBS (CBS in this case) unit is enough to generate a distinguishable signal, superior to the sensitivity of BiFC/TriFC RNA tracking platforms. These facts poise the intermolecular-allosteric-switch-based Cas6FC platform ideal for intracellularly tracking the location and abundance of target RNAs.

The authenticity of the signal generated from the Cas6FC system was verified by *in situ* RNA hybridization. The specificity of Cas6FC platform was interrogated and confirmed from two aspects: 1. *Ec*CBS is both structure- and sequence-specific for VN-d*Ec*Cas6-VC binding; 2. the *Ec*CBS motif for *Ec*Cas6 is unique without overlapping with the RNA sequences in the transcriptomes of multiple mammalian species. These properties preclude potential off-target effects to the most extent.

Recently, Cas9 and Cas13 were shown to be used to track RNAs of interest *in vivo*. Led by guide RNAs, Cas9/Cas13 could track genuine RNA free of RBS tags. However, they suffer from high background problem as other FE-type platforms. In this study, we again show that the CRISPR-Cas family is a precious resource for imaging RNAs. The Cas6FC reached almost free of background noise and needs only as minimal as one CBS tag for signal detection. One CBS is only 29nt long, which alleviates the workload of genetic manipulation. Moreover, a tag as small as one CBS reduces the risk of structural/locational alteration of an RNA target.

The Cas6FC RNA tracking platform could be further developed or optimized in a couple of ways: (i) many microbe species own Cas6-like enzymes and they could recognize different CBS from each other, enriching the toolbox of Cas6FC; (ii) various FPs with diverse excitation and emission spectrum could be examined for their amenability for Cas6FC; (iii) it is conceivable that simultaneous application of different Cas6-based reporters with different colors of FP could track multiple target RNAs in the same cell; in such a case, study of RNA–RNA interaction could be possible.

## Supplementary Material

gkac014_Supplemental_Files

## Notes

Present address: Chun-Yu Han, Gene Editing Research Center, Hebei University of Science and Technology, Shijiazhuang, Hebei 050018, PR China.
