# Correction to ‘A Cas6-based RNA tracking platform functioning in a fluorescence-activation mode’

**DOI:** 10.1093/nar/gkac111

**Published:** 2022-02-12

Nucleic Acids Research, gkac014, https://doi.org/10.1093/nar/gkac014

The authors have found that errors were introduced in Figures 4A and 4F during image assembly.

All the other figures are correct.

Figure 4A: the published figure shows the result of a mutant CdCBS (Corynebacterium diphtheriae) (C3G) which is irrelevant to the Figure. The panel has been substituted for the correct one in the new Figure 4A provided below.

Figure 4F: panel EcCBS_A22U was accidently duplicated from Figure 4E panel EcCBS_C14G|A15U. The correct panel is provided in the new Figure 4F below.

The corrections do not affect the results, discussion and conclusions presented in the article. The original published version has been updated.


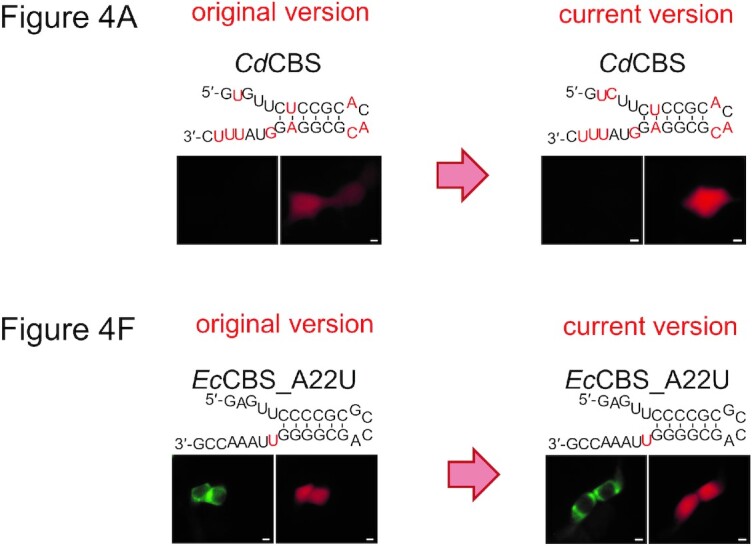



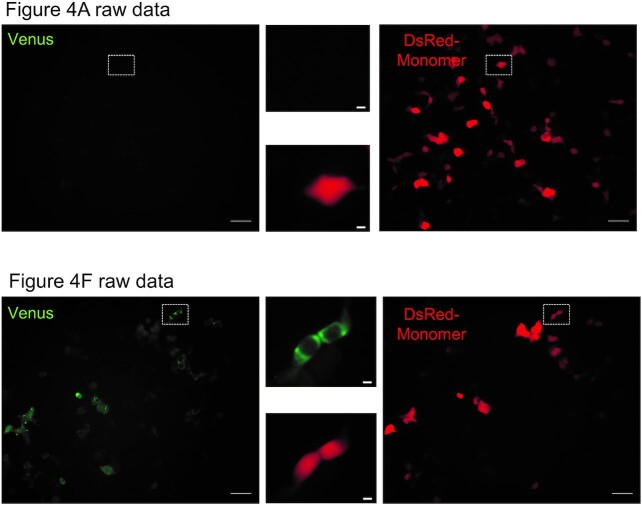



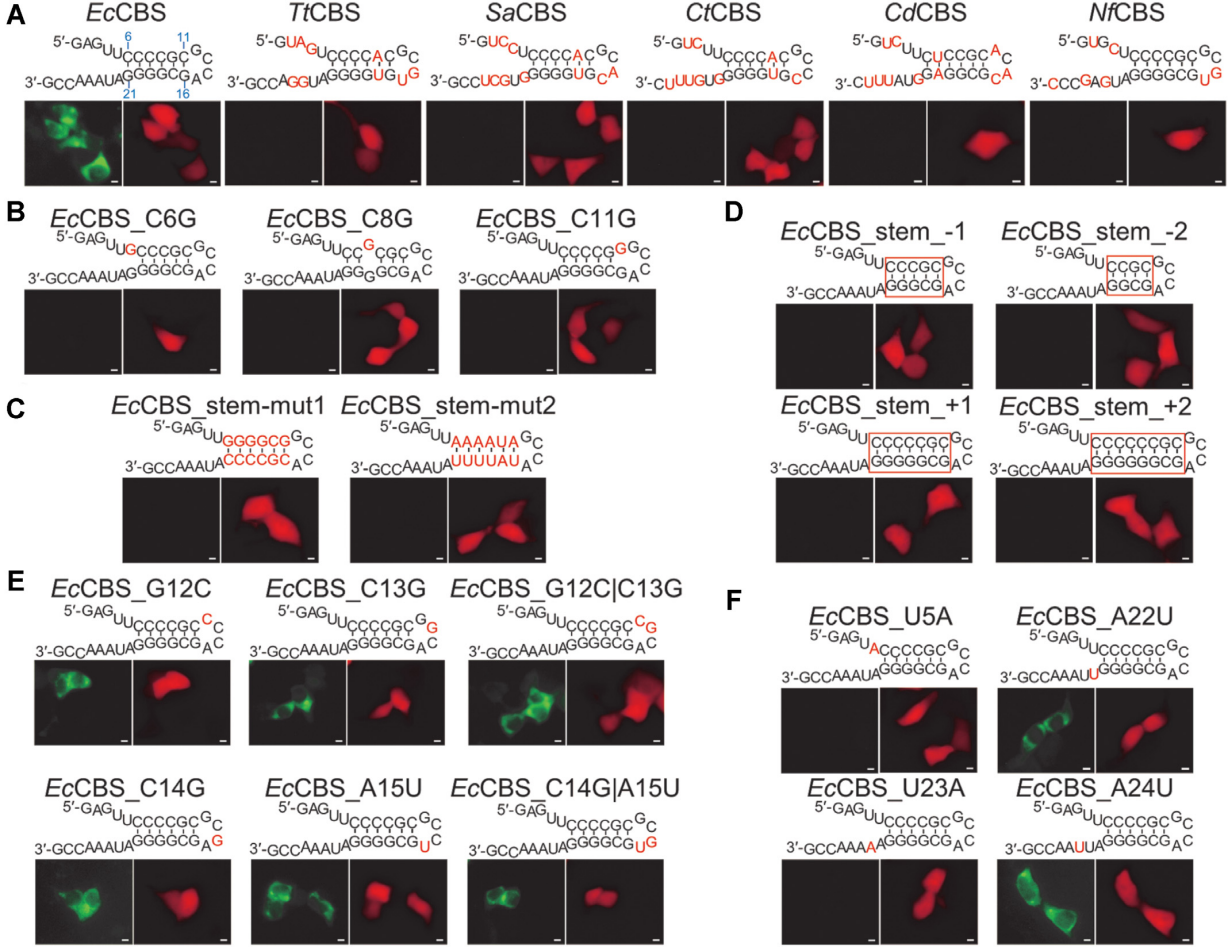


**New Figure 4**. The specificity of the Cas6FC platform. (**A**) Interactions between VN-dEcCas6-VC and EcCas6 cognate binding sites (CBSes) were examined in live cells. The nucleotides different from EcCBS on the cognate CBSes were highlighted in red. Tt for Thermus thermophilus; Sa for Salinispora Arenicola; Ct for Chlorobium tepidum; Cd for Corynebacterium diphtheriae. Nf for Nocardia farcinica. (**B**) The influences of stem-bulge mutations in EcCBS on VN-dEcCas6-VC recognition were examined. (**C**) The influences of transition and transversion mutations of EcCBS’s stem-localized nucleotides on VN-dEcCas6-VC recognition were examined. (**D**) The influences of stem length of EcCBS on VN-dEcCas6-VC recognition were examined. (**E**) The influences of loop-localized nucleotides mutations in EcCBS on VN-dEcCas6-VC recognition were examined. (**F**) The influences of non-stem-loop interacting nucleotide mutations in EcCBS on VN-dEcCas6-VC recognition were examined. The mutated nucleotides were highlighted in red. Scale bar, 5 μm. Representative pictures from three independent experiments were shown. The dosages of plasmids used were listed in Table 1.

