# Supplementary material for: A Cas6-based RNA tracking platform functioning in a fluorescence-activation mode
Source: Nucleic Acids Res. 2022 Jan 21;50(8):e46. doi: 10.1093/nar/gkac014 (PMC9071499; doi:10.1093/nar/gkac014)
Supplement: gkac014_Supplemental_Files [file gkac014_supplemental_files.zip › Supplementary Figures_2021-12-9.docx]

Supplementary Data to

A Cas6-based RNA tracking platform functioning in a fluorescence-activation mode

Feng Gao^1^, Ke Zheng^1^, You-Bo Li^2^, Feng Jiang^1^, Chun-Yu Han^1,^*

^1^Gene Editing Research Center, Hebei University of Science and Technology, Shijiazhuang, Hebei 050018, China.

^2^Hebei University, Baoding, Hebei 071000, China

^*^Corresponding author: hanchunyu@hebust.edu.cn

Supplementary Figure S1. *Ec*Cas6-GK construct

Supplementary Figure S2. d*Ec*Cas6-GK construct

Supplementary Figure S3. *Ec*Cas6-EGFP-N1 construct

Supplementary Figure S4. d*Ec*Cas6-EGFP-N1 construct

Supplementary Figure S5. CBS-EGFP-N1 construct

Supplementary Figure S6. Rm-16xCBS-Lin28-C1 construct

Supplementary Figure S7. VN-d*Ec*Cas6-VC-GK construct

Supplementary Figure S8. Rm-20×CBS-C1 construct

Supplementary Figure S9. Actin-GK construct

Supplementary Figure S10. Actin-1×CBS-GK construct

Supplementary Figure S11. Actin-2×CBS-GK construct

Supplementary Figure S12. Actin-4×CBS-GK construct

Supplementary Figure S13. Actin-8×CBS-GK construct

Supplementary Figure S14. Actin-16×CBS-GK construct

Supplementary Figure S15. hTERC-16×CBS-GK construct

Supplementary Figure S16. U6-1×CBS construct

Supplementary Figure S17. GK construct

Supplementary Figure S18. Rm-4×CBS variants series constructs

Supplementary Figure S19. CBS-binding induces an allosteric change of Cas6

Supplementary Figure S20. Tests of diverse linkers on Cas6FC RNA tracking

Supplementary Figure S21. sfGFP-based Cas6FC vs Venus-based Cas6FC RNA tracking

Supplementary Figure S22. Tests of diverse split-complementation strategies of venus for Cas6FC

Supplementary Figure S23. Applications of Cas6FC RNA tracking system in Hela and COS-7 cell lines.

Supplementary Figure S24. The nucleotides of CBS interacting with Cas6

**Supplementary Figure S1**


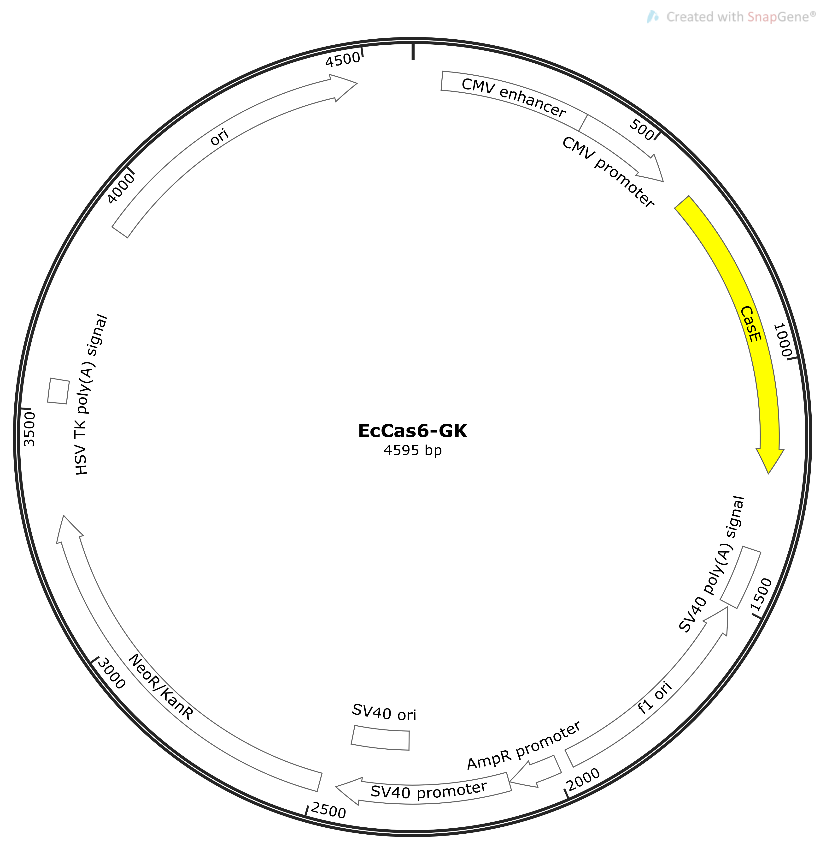


**Supplementary Figure S1.** *Ec*Cas6-GK construct

The coding sequence of *EcCas6* is marked in yellow.

GGTTTAGTGAACCGTCAGATCCGCTAGCGCTACCGGACTCAGATCTCGAGGCCACCATGtatctcagtaaagtcatcattgccagggcctggagcagggatctttaccaacttcaccagggattatggcatttatttccaaacagaccggatgctgctcgtgattttctttttcatgttgagaagcgaaacacaccagaaggctgtcatgttttattgcagtcagcgcaaatgcctgtttcaactgccgttgcgacagtcattaaaactaaacaggttgaatttcaacttcaggttggtgttccactctattttcggcttcgggcaaatccgatcaaaactattctcgacaatcaaaagcgcctggacagtaaagggaatattaaacgctgtcgggttccgttaataaaagaagcagaacaaatcgcgtggttgcaacgtaaattgggcaatgcggcgcgcgttgaagatgtgcatcccatatcggaacggccacagtatttttctggtgatggtaaaagtggaaagatccaaacggtttgctttgaaggtgtgctcaccatcaacgacgcgccagcgttaatagatcttgtacagcaaggtattgggccagctaaatcgatgggatgtggcttgctatctttggctccactgtgaGGTACCGCGGGCCCGGGATCCATATGTAAGTAAGTAAGCGGCCGCGACTCTAGATCATAATCAGCCATACCACATTTGTAGAGGTTTTACTTGCTTTAAAAAACCTCCCACACCTCCCCCTGAACCTGAAACATAAAATGAATGCAATTGTTGTTGTT

**Supplementary Figure S2**


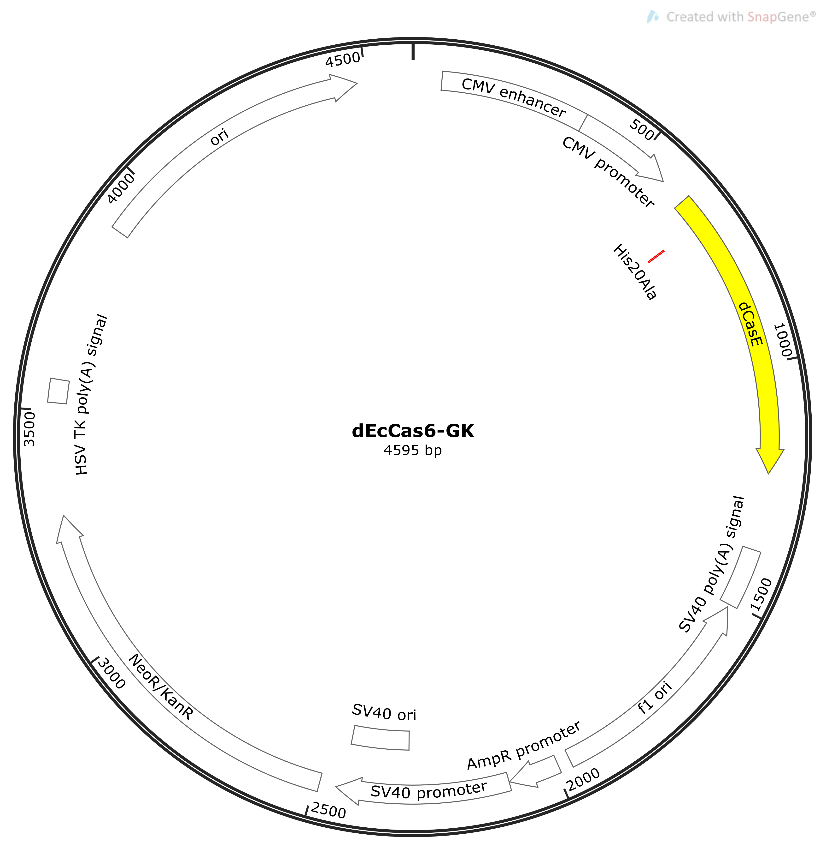


**Supplementary Figure S2.** d*Ec*Cas6-GK construct

The coding sequence of *dEcCas6* is marked in yellow. The mutation site His20Ala is marked in red.

GGTTTAGTGAACCGTCAGATCCGCTAGCGCTACCGGACTCAGATCTCGAGGCCACCATGtatctcagtaaagtcatcattgccagggcctggagcagggatctttaccaacttgcccagggattatggcatttatttccaaacagaccggatgctgctcgtgattttctttttcatgttgagaagcgaaacacaccagaaggctgtcatgttttattgcagtcagcgcaaatgcctgtttcaactgccgttgcgacagtcattaaaactaaacaggttgaatttcaacttcaggttggtgttccactctattttcggcttcgggcaaatccgatcaaaactattctcgacaatcaaaagcgcctggacagtaaagggaatattaaacgctgtcgggttccgttaataaaagaagcagaacaaatcgcgtggttgcaacgtaaattgggcaatgcggcgcgcgttgaagatgtgcatcccatatcggaacggccacagtatttttctggtgatggtaaaagtggaaagatccaaacggtttgctttgaaggtgtgctcaccatcaacgacgcgccagcgttaatagatcttgtacagcaaggtattgggccagctaaatcgatgggatgtggcttgctatctttggctccactgtgaGGTACCGCGGGCCCGGGATCCATATGTAAGTAAGTAAGCGGCCGCGACTCTAGATCATAATCAGCCATACCACATTTGTAGAGGTTTTACTTGCTTTAAAAAACCTCCCACACCTCCCCCTGAACCTGAAACATAAAATGAATGCAATTGTTGTTGTT

**Supplementary Figure S3**


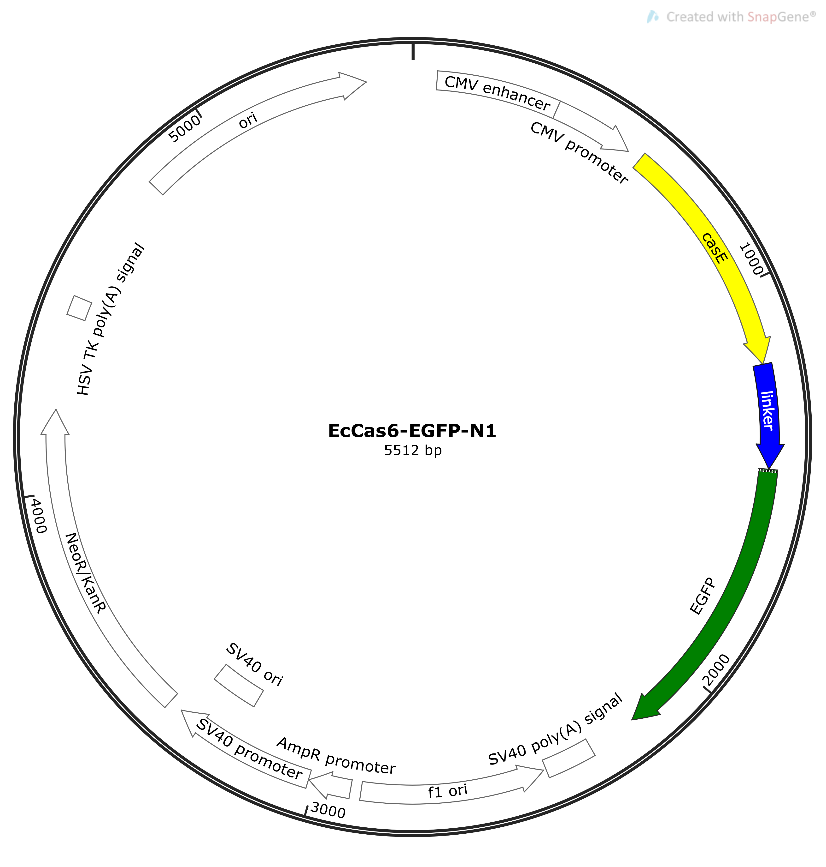


**Supplementary Figure S3.** *Ec*Cas6-EGFP-N1 construct.

The coding sequences of *EcCas6*, linker, and *EGFP* are shown in yellow, blue, and green, respectively.

ATGGTGtatctcagtaaagtcatcattgccagggcctggagcagggatctttaccaacttcaccagggattatggcatttatttccaaacagaccggatgctgctcgtgattttctttttcatgttgagaagcgaaacacaccagaaggctgtcatgttttattgcagtcagcgcaaatgcctgtttcaactgccgttgcgacagtcattaaaactaaacaggttgaatttcaacttcaggttggtgttccactctattttcggcttcgggcaaatccgatcaaaactattctcgacaatcaaaagcgcctggacagtaaagggaatattaaacgctgtcgggttccgttaataaaagaagcagaacaaatcgcgtggttgcaacgtaaattgggcaatgcggcgcgcgttgaagatgtgcatcccatatcggaacggccacagtatttttctggtgatggtaaaagtggaaagatccaaacggtttgctttgaaggtgtgctcaccatcaacgacgcgccagcgttaatagatcttgtacagcaaggtattgggccagctaaatcgatgggatgtggcttgctatctttggctccactgCTCGAGGGAGGCGGAGGCGGAAGCGGCGGAGGAGGAAGCGGCGGAGGCGGAAGCGGCGGAATTCAGAGCGGCGGAGGAGGAAGCGGCGGAGGAGGCAGCGGCGGAGGAGGAAGCGGGTCGACGGGCGGAGGCGGAAGCGGCGGAGGAGGCAGCGGCGGAGGCGGAAGCGGGGTACCTGGCGGAGGAGGCAGCGGCGGAGGAGGAAGCGGCGGAGGAGGAAGCGGCGGAGGAGGCAGCGGGGATCCACCGGTCGCCACCATGgtgagcaagggcgaggagctgttcaccggggtggtgcccatcctggtcgagctggacggcgacgtaaacggccacaagttcagcgtgtccggcgagggcgagggcgatgccacctacggcaagctgaccctgaagttcatctgcaccaccggcaagctgcccgtgccctggcccaccctcgtgaccaccctgacctacggcgtgcagtgcttcagccgctaccccgaccacatgaagcagcacgacttcttcaagtccgccatgcccgaaggctacgtccaggagcgcaccatcttcttcaaggacgacggcaactacaagacccgcgccgaggtgaagttcgagggcgacaccctggtgaaccgcatcgagctgaagggcatcgacttcaaggaggacggcaacatcctggggcacaagctggagtacaactacaacagccacaacgtctatatcatggccgacaagcagaagaacggcatcaaggtgaacttcaagatccgccacaacatcgaggacggcagcgtgcagctcgccgaccactaccagcagaacacccccatcggcgacggccccgtgctgctgcccgacaaccactacctgagcacccagtccgccctgagcaaagaccccaacgagaagcgcgatcacatggtcctgctggagttcgtgaccgccgccgggatcactctcggcatggacgagctgtacaagtaa

Their deduced amino acid sequences are:

MVYLSKVIIARAWSRDLYQLHQGLWHLFPNRPDAARDFLFHVEKRNTPEGCHVLLQSAQMPVSTAVATVIKTKQVEFQLQVGVPLYFRLRANPIKTILDNQKRLDSKGNIKRCRVPLIKEAEQIAWLQRKLGNAARVEDVHPISERPQYFSGDGKSGKIQTVCFEGVLTINDAPALIDLVQQGIGPAKSMGCGLLSLAPLLEGGGGGSGGGGSGGGGSGGIQSGGGGSGGGGSGGGGSGSTGGGGSGGGGSGGGGSGVPGGGGSGGGGSGGGGSGGGGSGDPPVATMVSKGEELFTGVVPILVELDGDVNGHKFSVSGEGEGDATYGKLTLKFICTTGKLPVPWPTLVTTLTYGVQCFSRYPDHMKQHDFFKSAMPEGYVQERTIFFKDDGNYKTRAEVKFEGDTLVNRIELKGIDFKEDGNILGHKLEYNYNSHNVYIMADKQKNGIKVNFKIRHNIEDGSVQLADHYQQNTPIGDGPVLLPDNHYLSTQSALSKDPNEKRDHMVLLEFVTAAGITLGMDELYK*

**Supplementary Figure S4**


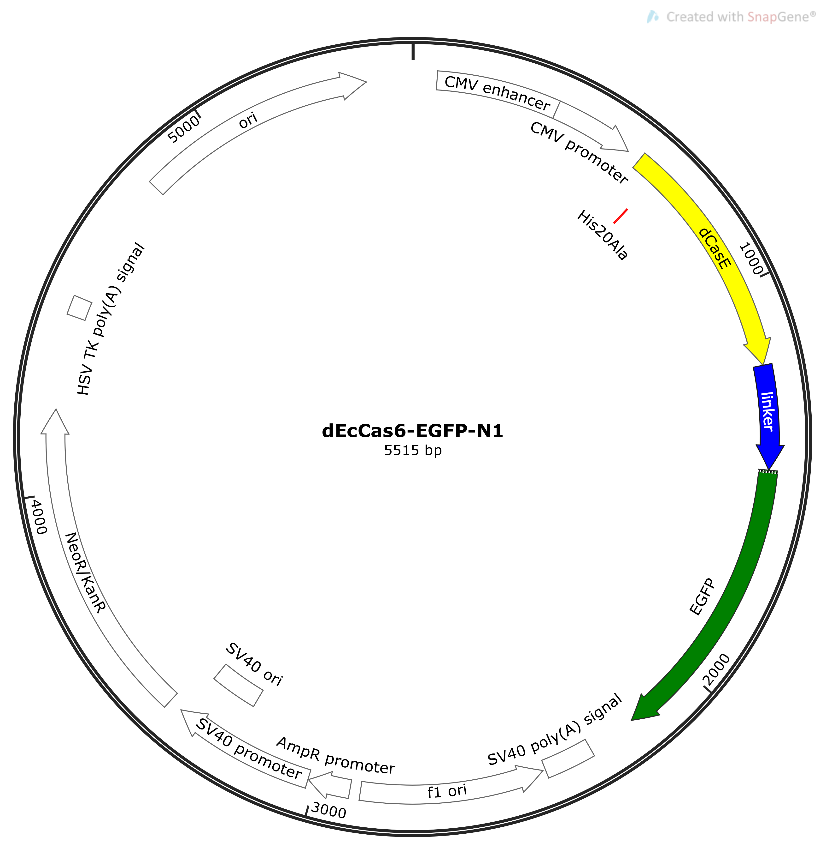


**Supplementary Figure S4.** d*Ec*Cas6-EGFP-N1 construct.

The coding sequences of *dEcCas6*, peptide linker, and *EGFP* are shown in yellow, blue, and green, respectively. The mutation site His20Ala is marked in red.

ATGGTGtatctcagtaaagtcatcattgccagggcctggagcagggatctttaccaacttgcccagggattatggcatttatttccaaacagaccggatgctgctcgtgattttctttttcatgttgagaagcgaaacacaccagaaggctgtcatgttttattgcagtcagcgcaaatgcctgtttcaactgccgttgcgacagtcattaaaactaaacaggttgaatttcaacttcaggttggtgttccactctattttcggcttcgggcaaatccgatcaaaactattctcgacaatcaaaagcgcctggacagtaaagggaatattaaacgctgtcgggttccgttaataaaagaagcagaacaaatcgcgtggttgcaacgtaaattgggcaatgcggcgcgcgttgaagatgtgcatcccatatcggaacggccacagtatttttctggtgatggtaaaagtggaaagatccaaacggtttgctttgaaggtgtgctcaccatcaacgacgcgccagcgttaatagatcttgtacagcaaggtattgggccagctaaatcgatgggatgtggcttgctatctttggctccactgCTCGAGGGAGGCGGAGGCGGAAGCGGCGGAGGAGGAAGCGGCGGAGGCGGAAGCGGCGGAATTCAGAGCGGCGGAGGAGGAAGCGGCGGAGGAGGCAGCGGCGGAGGAGGAAGCGGGTCGACGGGCGGAGGCGGAAGCGGCGGAGGAGGCAGCGGCGGAGGCGGAAGCGGGGTACCTGGCGGAGGAGGCAGCGGCGGAGGAGGAAGCGGCGGAGGAGGAAGCGGCGGAGGAGGCAGCGGGGATCCACCGGTCGCCACCATGgtgagcaagggcgaggagctgttcaccggggtggtgcccatcctggtcgagctggacggcgacgtaaacggccacaagttcagcgtgtccggcgagggcgagggcgatgccacctacggcaagctgaccctgaagttcatctgcaccaccggcaagctgcccgtgccctggcccaccctcgtgaccaccctgacctacggcgtgcagtgcttcagccgctaccccgaccacatgaagcagcacgacttcttcaagtccgccatgcccgaaggctacgtccaggagcgcaccatcttcttcaaggacgacggcaactacaagacccgcgccgaggtgaagttcgagggcgacaccctggtgaaccgcatcgagctgaagggcatcgacttcaaggaggacggcaacatcctggggcacaagctggagtacaactacaacagccacaacgtctatatcatggccgacaagcagaagaacggcatcaaggtgaacttcaagatccgccacaacatcgaggacggcagcgtgcagctcgccgaccactaccagcagaacacccccatcggcgacggccccgtgctgctgcccgacaaccactacctgagcacccagtccgccctgagcaaagaccccaacgagaagcgcgatcacatggtcctgctggagttcgtgaccgccgccgggatcactctcggcatggacgagctgtacaagtaa

Their deduced amino acid sequences are:

MVYLSKVIIARAWSRDLYQLAQGLWHLFPNRPDAARDFLFHVEKRNTPEGCHVLLQSAQMPVSTAVATVIKTKQVEFQLQVGVPLYFRLRANPIKTILDNQKRLDSKGNIKRCRVPLIKEAEQIAWLQRKLGNAARVEDVHPISERPQYFSGDGKSGKIQTVCFEGVLTINDAPALIDLVQQGIGPAKSMGCGLLSLAPLLEGGGGGSGGGGSGGGGSGGIQSGGGGSGGGGSGGGGSGSTGGGGSGGGGSGGGGSGVPGGGGSGGGGSGGGGSGGGGSGDPPVATMVSKGEELFTGVVPILVELDGDVNGHKFSVSGEGEGDATYGKLTLKFICTTGKLPVPWPTLVTTLTYGVQCFSRYPDHMKQHDFFKSAMPEGYVQERTIFFKDDGNYKTRAEVKFEGDTLVNRIELKGIDFKEDGNILGHKLEYNYNSHNVYIMADKQKNGIKVNFKIRHNIEDGSVQLADHYQQNTPIGDGPVLLPDNHYLSTQSALSKDPNEKRDHMVLLEFVTAAGITLGMDELYK*

**Supplementary Figure S5**

**Supplementary Figure S5.** CBS-EGFP-N1 construct.

The nucleotide sequences of CBS and *EGFP* are marked in blue and green, respectively.

GGTTTAGTGAACCGTCAGATCCGCTAGCGCTACCGGACTCAGATCTCGACCGAGTTCCCCGCGCCAGCGGGGATAAACCGCGCCAGCTTCGAATTCTGCAGTCGACGGTACCGCGGGCCCGGGATCCACCGGTCGCCACCATGgtgagcaagggcgaggagctgttcaccggggtggtgcccatcctggtcgagctggacggcgacgtaaacggccacaagttcagcgtgtccggcgagggcgagggcgatgccacctacggcaagctgaccctgaagttcatctgcaccaccggcaagctgcccgtgccctggcccaccctcgtgaccaccctgacctacggcgtgcagtgcttcagccgctaccccgaccacatgaagcagcacgacttcttcaagtccgccatgcccgaaggctacgtccaggagcgcaccatcttcttcaaggacgacggcaactacaagacccgcgccgaggtgaagttcgagggcgacaccctggtgaaccgcatcgagctgaagggcatcgacttcaaggaggacggcaacatcctggggcacaagctggagtacaactacaacagccacaacgtctatatcatggccgacaagcagaagaacggcatcaaggtgaacttcaagatccgccacaacatcgaggacggcagcgtgcagctcgccgaccactaccagcagaacacccccatcggcgacggccccgtgctgctgcccgacaaccactacctgagcacccagtccgccctgagcaaagaccccaacgagaagcgcgatcacatggtcctgctggagttcgtgaccgccgccgggatcactctcggcatggacgagctgtacaagtaaAGCGGCCGCGACTCTAGATCATAATCAGCCATACCACATTTGTAGAGGTTTTACTTGCTTTAAAAAACCTCCCACACCTCCCCCTGAACCTGAAACATAAAATGAATGCAATTGTTGTTGTT

**Supplementary Figure S6**

**Supplementary Figure S6.** Rm-16×CBS-Lin28-C1 construct.

The nucleotide sequences of *DsRed-Monomer* (abbreviated as *Rm*) gene, CBS, and Lin28 are marked in red, blue, and pink, respectively.

GGTTTAGTGAACCGTCAGATCCGCTAGCACCGGTCGCCACCATGgacaacaccgaggacgtcatcaaggagttcatgcagttcaaggtgcgcatggagggctccgtgaacggccactacttcgagatcgagggcgagggcgagggcaagccctacgagggcacccagaccgccaagctgcaggtgaccaagggcggccccctgcccttcgcctgggacatcctgtccccccagttccagtacggctccaaggcctacgtgaagcaccccgccgacatccccgactacatgaagctgtccttccccgagggcttcacctgggagcgctccatgaacttcgaggacggcggcgtggtggaggtgcagcaggactcctccctgcaggacggcaccttcatctacaaggtgaagttcaagggcgtgaacttccccgccgacggccccgtaatgcagaagaagactgccggctgggagccctccaccgagaagctgtacccccaggacggcgtgctgaagggcgagatctcccacgccctgaagctgaaggacggcggccactacacctgcgacttcaagaccgtgtacaaggccaagaagcccgtgcagctgcccggcaaccactacgtggactccaagctggacatcaccaaccacaacgaggactacaccgtggtggagcagtacgagcacgccgaggcccgccactccggctcccagtccggactcagatctcgactttgaGCGCTACCGGACTCAGATCTCGACCGAGTTCCCCGCGCCAGCGGGGATAAACCGCGCCAGCTTCGAATTCTGCAGTCGAAGAGTTCCCCGCGCCAGCGGGGATAAACCCGATCCACCGGTATTTATCTCGACCGAGTTCCCCGCGCCAGCGGGGATAAACCGCGCCAGCTTCGAATTCGAGTTCCCCGCGCCAGCGGGGATAAACCCGATCCACCGGTATTTATCTCGACCGAGTTCCCCGCGCCAGCGGGGATAAACCGCGCCAGCTTCGAATTCTGCAGTCGAAGAGTTCCCCGCGCCAGCGGGGATAAACCCGATCCACCGGTATTTATCTCGACCGAGTTCCCCGCGCCAGCGGGGATAAACCGCGCCAGCTTCGAATTCGAGTTCCCCGCGCCAGCGGGGATAAACCCGATCCACCGGTATTTATCTCGACCGAGTTCCCCGCGCCAGCGGGGATAAACCGCGCCAGCTTCGAATTCTGCAGTCGAAGAGTTCCCCGCGCCAGCGGGGATAAACCCGATCCACCGGTATTTATCTCGACCGAGTTCCCCGCGCCAGCGGGGATAAACCGCGCCAGCTTCGAATTCGAGTTCCCCGCGCCAGCGGGGATAAACCCGATCCACCGGTATTTATCTCGACCGAGTTCCCCGCGCCAGCGGGGATAAACCGCGCCAGCTTCGAATTCTGCAGTCGAAGAGTTCCCCGCGCCAGCGGGGATAAACCCGATCCACCGGTATTTATCTCGACCGAGTTCCCCGCGCCAGCGGGGATAAACCGCGCCAGCTTCGAATTCGAGTTCCCCGCGCCAGCGGGGATAAACCCGATCCACCGGTATTTATCTCGAGTTTGATAAGGAAaaccctccatcccttgttcccaacctcctaagtcaagaccattaccatttctttctttttttggtggggggggggtggatggagtctcactgtgtcgcccaggctggagtgcagtggcatgatcggctcactgcagcctctgcctcttgggttcaagtgattctcctgcctcagcctcctgagtagctgggatttcaggcacccgccacactcagctaatttttgtatttttagtagagacggggtttcaccatgttgtccaggctggtctggaactcctgacctcaggtgatctgcccaccttggcttcccaaagtgctgggattacaggcatgagccaccatgctgggccaaccatttcttggtgtattcatgccaaacacttaagacactgctgtagcctgggcgcagtggctcacacctgtagtcccagcactttggaaggctgaggcgggcgaatcacaaggtcaggagttcaaaaccatcctggccaacacggtgaaaccccgtctctgctgaaatacaaaaaaattagccaggtgtggtggcgcatacctgtggtcccggctattcaggaggctgaggcgggggaatcgctttaacctggaaggcggaggttgcagtgagctgagatcgcaccactgcactccagcctggttacagagcaagactctgtctcgaaaaaaaaaaaaaaaacaaaaaaacaacaaaaaaaaaaccacactactgtattttggatggatcaaacctccttaaAAGCTTGGGGGGATCCACCGGATCTAGATAACTGATCATAATCAGCCATACCACATTTGTAGAGGTTTTACTTGCTTTAAAAAACCTCCCACACCTCCCCCTGAACCTGAAACATAAAATGAATGCAATTGTTGTTGTT

**Supplementary Figure S7**


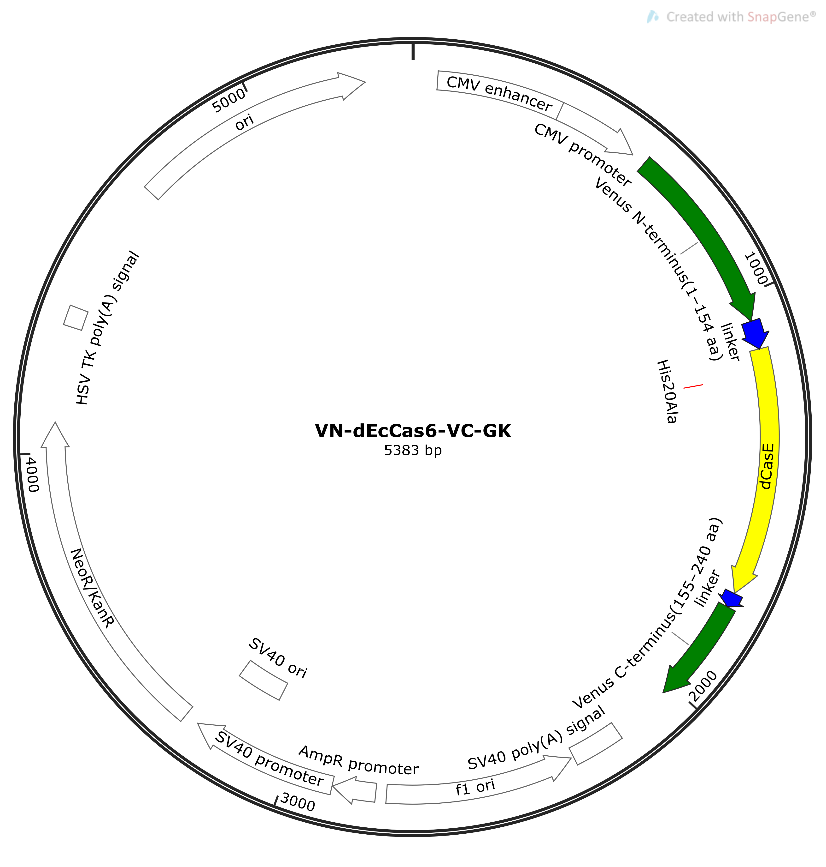


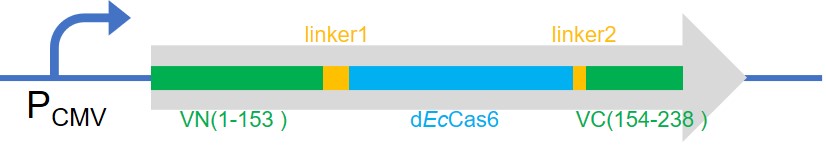


**Supplementary Figure S7.** VN-d*Ec*Cas6-VC-GK construct.

The nucleotide sequences of the *Venus* N-terminus (1‒153 aa) and C-terminus (154‒238 aa), linkers, *dEcCas6*, and the mutation site His20Ala are marked in green, blue, yellow, and red, respectively.

ATGgtgagcaagggcgaggagctgttcaccggggtggtgcccatcctggtcgagctggacggcgacgtaaacggccacaagttcagcgtgtccggcgagggcgagggcgatgccacctacggcaagctgaccctgaagctgatctgcaccaccggcaagctgcccgtgccctggcccaccctcgtgaccaccctgggctacggcctgcagtgcttcgcccgctaccccgaccacatgaagcagcacgacttcttcaagtccgccatgcccgaaggctacgtccaggagcgcaccatcttcttcaaggacgacggcaactacaagacccgcgccgaggtgaagttcgagggcgacaccctggtgaaccgcatcgagctgaagggcatcgacttcaaggaggacggcaacatcctggggcacaagctggagtacaactacaacagccacaacgtctatatcaccCTCGAGAGACCTGCTTGTAAAATTCCAAACGACCTGAAGCAGAAAGTGATGAACCACAAGCTTGCCACCATGGTGtatctcagtaaagtcatcattgccagggcctggagcagggatctttaccaacttgcccagggattatggcatttatttccaaacagaccggatgctgctcgtgattttctttttcatgttgagaagcgaaacacaccagaaggctgtcatgttttattgcagtcagcgcaaatgcctgtttcaactgccgttgcgacagtcattaaaactaaacaggttgaatttcaacttcaggttggtgttccactctattttcggcttcgggcaaatccgatcaaaactattctcgacaatcaaaagcgcctggacagtaaagggaatattaaacgctgtcgggttccgttaataaaagaagcagaacaaatcgcgtggttgcaacgtaaattgggcaatgcggcgcgcgttgaagatgtgcatcccatatcggaacggccacagtatttttctggtgatggtaaaagtggaaagatccaaacggtttgctttgaaggtgtgctcaccatcaacgacgcgccagcgttaatagatcttgtacagcaaggtattgggccagctaaatcgatgggatgtggcttgctatctttggctccactGCTGCAGTCGACGACCTGCACAGCTGGCGCTGAATTCgccgacaagcagaagaacggcatcaaggccaacttcaagatccgccacaacatcgaggacggcggcgtgcagctcgccgaccactaccagcagaacacccccatcggcgacggccccgtgctgctgcccgacaaccactacctgagctaccagtccgccctgagcaaagaccccaacgagaagcgcgatcacatggtcctgctggagttcgtgaccgccgccgggatcactctcggcatggacgagctgtacaagtaa

Their deduced amino acid sequences are:

MVSKGEELFTGVVPILVELDGDVNGHKFSVSGEGEGDATYGKLTLKLICTTGKLPVPWPTLVTTLGYGLQCFARYPDHMKQHDFFKSAMPEGYVQERTIFFKDDGNYKTRAEVKFEGDTLVNRIELKGIDFKEDGNILGHKLEYNYNSHNVYITLERPACKIPNDLKQKVMNHKLATMVYLSKVIIARAWSRDLYQLAQGLWHLFPNRPDAARDFLFHVEKRNTPEGCHVLLQSAQMPVSTAVATVIKTKQVEFQLQVGVPLYFRLRANPIKTILDNQKRLDSKGNIKRCRVPLIKEAEQIAWLQRKLGNAARVEDVHPISERPQYFSGDGKSGKIQTVCFEGVLTINDAPALIDLVQQGIGPAKSMGCGLLSLAPLLQSTTCTAGAEFADKQKNGIKANFKIRHNIEDGGVQLADHYQQNTPIGDGPVLLPDNHYLSYQSALSKDPNEKRDHMVLLEFVTAAGITLGMDELYK*

**Supplementary Figure S8**

**Supplementary Figure S8.** Rm-20×CBS-C1 construct.

The nucleotide sequences of *DsRed-monomer* (abbreviated as *Rm*) gene and CBS are marked in red and blue, respectively.

GGTTTAGTGAACCGTCAGATCCGCTAGCACCGGTCGCCACCATGgacaacaccgaggacgtcatcaaggagttcatgcagttcaaggtgcgcatggagggctccgtgaacggccactacttcgagatcgagggcgagggcgagggcaagccctacgagggcacccagaccgccaagctgcaggtgaccaagggcggccccctgcccttcgcctgggacatcctgtccccccagttccagtacggctccaaggcctacgtgaagcaccccgccgacatccccgactacatgaagctgtccttccccgagggcttcacctgggagcgctccatgaacttcgaggacggcggcgtggtggaggtgcagcaggactcctccctgcaggacggcaccttcatctacaaggtgaagttcaagggcgtgaacttccccgccgacggccccgtaatgcagaagaagactgccggctgggagccctccaccgagaagctgtacccccaggacggcgtgctgaagggcgagatctcccacgccctgaagctgaaggacggcggccactacacctgcgacttcaagaccgtgtacaaggccaagaagcccgtgcagctgcccggcaaccactacgtggactccaagctggacatcaccaaccacaacgaggactacaccgtggtggagcagtacgagcacgccgaggcccgccactccggctcccagtccggactcagatctcgactttgaGCGCTACCGGACTCAGATCTCGACCGAGTTCCCCGCGCCAGCGGGGATAAACCGCGCCAGCTTCGAATTCTGCAGTCGAAGAGTTCCCCGCGCCAGCGGGGATAAACCCGATCCACCGGTATTTATCTCGACCGAGTTCCCCGCGCCAGCGGGGATAAACCGCGCCAGCTTCGAATTCGAGTTCCCCGCGCCAGCGGGGATAAACCCGATCCACCGGTATTTATCTCGACCGAGTTCCCCGCGCCAGCGGGGATAAACCGCGCCAGCTTCGAATTCTGCAGTCGAAGAGTTCCCCGCGCCAGCGGGGATAAACCCGATCCACCGGTATTTATCTCGACCGAGTTCCCCGCGCCAGCGGGGATAAACCGCGCCAGCTTCGAATTCGAGTTCCCCGCGCCAGCGGGGATAAACCCGATCCACCGGTATTTATCTCGACCGAGTTCCCCGCGCCAGCGGGGATAAACCGCGCCAGCTTCGAATTCTGCAGTCGAAGAGTTCCCCGCGCCAGCGGGGATAAACCCGATCCACCGGTATTTATCTCGACCGAGTTCCCCGCGCCAGCGGGGATAAACCGCGCCAGCTTCGAATTCGAGTTCCCCGCGCCAGCGGGGATAAACCCGATCCACCGGTATTTATCTCGACCGAGTTCCCCGCGCCAGCGGGGATAAACCGCGCCAGCTTCGAATTCTGCAGTCGAAGAGTTCCCCGCGCCAGCGGGGATAAACCCGATCCACCGGTATTTATCTCGACCGAGTTCCCCGCGCCAGCGGGGATAAACCGCGCCAGCTTCGAATTCGAGTTCCCCGCGCCAGCGGGGATAAACCCGATCCACCGGTATTTATCTCGAGGGAAGCTTCGAATTCTGCAGTCGACCGAGTTCCCCGCGCCAGCGGGGATAAACCGCGCCAGCTTCGAATTCTGCAGTCGAAGAGTTCCCCGCGCCAGCGGGGATAAACCCGATCCACCGGTATTTATCTCGACCGAGTTCCCCGCGCCAGCGGGGATAAACCGCGCCAGCTTCGAATTCTGCAGTCGAAGAGTTCCCCGCGCCAGCGGGGATAAACCCGATCCACCGGTATTTATCTCGAGGGAAGCTTCGAATTCTGCAGTCGACGGTACCGCGGGCCCgGGATCCACCGGATCTAGATAACTGATCATAATCAGCCATACCACATTTGTAGAGGTTTTACTTGCTTTAAAAAACCTCCCACACCTCCCCCTGAACCTGAAACATAAAATGAATGCAATTGTTGTTGTT

**Supplementary Figure S9**

**Supplementary Figure S9.** Actin-GK construct.

The nucleotide sequence of *ACTB* gene is marked in orange.

GGTTTAGTGAACCGTCAGATCCGCTAGCGCCACCATGgatgatgatatcgccgcgctcgtcgtcgacaacggctccggcatgtgcaaggccggcttcgcgggcgacgatgccccccgggccgtcttcccctccatcgtggggcgccccaggcaccagggcgtgatggtgggcatgggtcagaaggattcctatgtgggcgacgaggcccagagcaagagaggcatcctcaccctgaagtaccccatcgagcacggcatcgtcaccaactgggacgacatggagaaaatctggcaccacaccttctacaatgagctgcgtgtggctcccgaggagcaccccgtgctgctgaccgaggcccccctgaaccccaaggccaaccgcgagaagatgacccagatcatgtttgagaccttcaacaccccagccatgtacgttgctatccaggctgtgctatccctgtacgcctctggccgtaccactggcatcgtgatggactccggtgacggggtcacccacactgtgcccatctacgaggggtatgccctcccccatgccatcctgcgtctggacctggctggccgggacctgactgactacctcatgaagatcctcaccgagcgcggctacagcttcaccaccacggccgagcgggaaatcgtgcgtgacattaaggagaagctgtgctacgtcgccctggacttcgagcaagagatggccacggctgcttccagctcctccctggagaagagctacgagctgcctgacggccaggtcatcaccattggcaatgagcggttccgctgccctgaggcactcttccagccttccttcctgggcatggagtcctgtggcatccacgaaactaccttcaactccatcatgaagtgtgacgtggacatccgcaaagacctgtacgccaacacagtgctgtctggcggcaccaccatgtaccctggcattgccgacaggatgcagaaggagatcactgccctggcacccagcacaatgaagatcaagatcattgctcctcctgagcgcaagtactccgtgtggatcggcggctccatcctggcctcgctgtccaccttccagcagatgtggatcagcaagcaggagtatgacgagtccggcccctccatcgtccaccgcaaatgcttctgaGATCTCGAGCTCAAGCTTCGAATTCTGCAGTCGACGGTACCGCGGGCCCGGGATCCATATGTAAGTAAGTAAGCGGCCGCGACTCTAGATCATAATCAGCCATACCACATTTGTAGAGGTTTTACTTGCTTTAAAAAACCTCCCACACCTCCCCCTGAACCTGAAACATAAAATGAATGCAATTGTTGTTGTT

**Supplementary Figure S10**

**Supplementary Figure S10.** Actin-1×CBS-GK construct.

The nucleotide sequences of *ACTB* and CBS are marked in orange and blue, respectively.

GGTTTAGTGAACCGTCAGATCCGCTAGCGCCACCATGgatgatgatatcgccgcgctcgtcgtcgacaacggctccggcatgtgcaaggccggcttcgcgggcgacgatgccccccgggccgtcttcccctccatcgtggggcgccccaggcaccagggcgtgatggtgggcatgggtcagaaggattcctatgtgggcgacgaggcccagagcaagagaggcatcctcaccctgaagtaccccatcgagcacggcatcgtcaccaactgggacgacatggagaaaatctggcaccacaccttctacaatgagctgcgtgtggctcccgaggagcaccccgtgctgctgaccgaggcccccctgaaccccaaggccaaccgcgagaagatgacccagatcatgtttgagaccttcaacaccccagccatgtacgttgctatccaggctgtgctatccctgtacgcctctggccgtaccactggcatcgtgatggactccggtgacggggtcacccacactgtgcccatctacgaggggtatgccctcccccatgccatcctgcgtctggacctggctggccgggacctgactgactacctcatgaagatcctcaccgagcgcggctacagcttcaccaccacggccgagcgggaaatcgtgcgtgacattaaggagaagctgtgctacgtcgccctggacttcgagcaagagatggccacggctgcttccagctcctccctggagaagagctacgagctgcctgacggccaggtcatcaccattggcaatgagcggttccgctgccctgaggcactcttccagccttccttcctgggcatggagtcctgtggcatccacgaaactaccttcaactccatcatgaagtgtgacgtggacatccgcaaagacctgtacgccaacacagtgctgtctggcggcaccaccatgtaccctggcattgccgacaggatgcagaaggagatcactgccctggcacccagcacaatgaagatcaagatcattgctcctcctgagcgcaagtactccgtgtggatcggcggctccatcctggcctcgctgtccaccttccagcagatgtggatcagcaagcaggagtatgacgagtccggcccctccatcgtccaccgcaaatgcttctgaGATCCGAGTTCCCCGCGCCAGCGGGGATAAACCGCTACTACCTCATTGATCCTGGCTTGATCCATATGTAAGTAAGTAAGCGGCCGCGACTCTAGATCATAATCAGCCATACCACATTTGTAGAGGTTTTACTTGCTTTAAAAAACCTCCCACACCTCCCCCTGAACCTGAAACATAAAATGAATGCAATTGTTGTTGTT

**Supplementary Figure S11**

**Supplementary Figure S11.** Actin-2×CBS-GK construct.

The nucleotide sequences of *ACTB* and CBS are marked in orange and blue, respectively.

GGTTTAGTGAACCGTCAGATCCGCTAGCGCCACCATGgatgatgatatcgccgcgctcgtcgtcgacaacggctccggcatgtgcaaggccggcttcgcgggcgacgatgccccccgggccgtcttcccctccatcgtggggcgccccaggcaccagggcgtgatggtgggcatgggtcagaaggattcctatgtgggcgacgaggcccagagcaagagaggcatcctcaccctgaagtaccccatcgagcacggcatcgtcaccaactgggacgacatggagaaaatctggcaccacaccttctacaatgagctgcgtgtggctcccgaggagcaccccgtgctgctgaccgaggcccccctgaaccccaaggccaaccgcgagaagatgacccagatcatgtttgagaccttcaacaccccagccatgtacgttgctatccaggctgtgctatccctgtacgcctctggccgtaccactggcatcgtgatggactccggtgacggggtcacccacactgtgcccatctacgaggggtatgccctcccccatgccatcctgcgtctggacctggctggccgggacctgactgactacctcatgaagatcctcaccgagcgcggctacagcttcaccaccacggccgagcgggaaatcgtgcgtgacattaaggagaagctgtgctacgtcgccctggacttcgagcaagagatggccacggctgcttccagctcctccctggagaagagctacgagctgcctgacggccaggtcatcaccattggcaatgagcggttccgctgccctgaggcactcttccagccttccttcctgggcatggagtcctgtggcatccacgaaactaccttcaactccatcatgaagtgtgacgtggacatccgcaaagacctgtacgccaacacagtgctgtctggcggcaccaccatgtaccctggcattgccgacaggatgcagaaggagatcactgccctggcacccagcacaatgaagatcaagatcattgctcctcctgagcgcaagtactccgtgtggatcggcggctccatcctggcctcgctgtccaccttccagcagatgtggatcagcaagcaggagtatgacgagtccggcccctccatcgtccaccgcaaatgcttctgaGATCTGACCGAGTTCCCCGCGCCAGCGGGGATAAACCGCGCCAGCTTCGCGACGAGTTCCCCGCGCCAGCGGGGATAAACCGCGCCGGATCCATATGTAAGTAAGTAAGCGGCCGCGACTCTAGATCATAATCAGCCATACCACATTTGTAGAGGTTTTACTTGCTTTAAAAAACCTCCCACACCTCCCCCTGAACCTGAAACATAAAATGAATGCAATTGTTGTTGTT

**Supplementary Figure S12**

**Supplementary Figure S12.** Actin-4×CBS-GK construct.

The nucleotide sequences of *ACTB* and CBS are marked in orange and blue, respectively.

GGTTTAGTGAACCGTCAGATCCGCTAGCGCCACCATGgatgatgatatcgccgcgctcgtcgtcgacaacggctccggcatgtgcaaggccggcttcgcgggcgacgatgccccccgggccgtcttcccctccatcgtggggcgccccaggcaccagggcgtgatggtgggcatgggtcagaaggattcctatgtgggcgacgaggcccagagcaagagaggcatcctcaccctgaagtaccccatcgagcacggcatcgtcaccaactgggacgacatggagaaaatctggcaccacaccttctacaatgagctgcgtgtggctcccgaggagcaccccgtgctgctgaccgaggcccccctgaaccccaaggccaaccgcgagaagatgacccagatcatgtttgagaccttcaacaccccagccatgtacgttgctatccaggctgtgctatccctgtacgcctctggccgtaccactggcatcgtgatggactccggtgacggggtcacccacactgtgcccatctacgaggggtatgccctcccccatgccatcctgcgtctggacctggctggccgggacctgactgactacctcatgaagatcctcaccgagcgcggctacagcttcaccaccacggccgagcgggaaatcgtgcgtgacattaaggagaagctgtgctacgtcgccctggacttcgagcaagagatggccacggctgcttccagctcctccctggagaagagctacgagctgcctgacggccaggtcatcaccattggcaatgagcggttccgctgccctgaggcactcttccagccttccttcctgggcatggagtcctgtggcatccacgaaactaccttcaactccatcatgaagtgtgacgtggacatccgcaaagacctgtacgccaacacagtgctgtctggcggcaccaccatgtaccctggcattgccgacaggatgcagaaggagatcactgccctggcacccagcacaatgaagatcaagatcattgctcctcctgagcgcaagtactccgtgtggatcggcggctccatcctggcctcgctgtccaccttccagcagatgtggatcagcaagcaggagtatgacgagtccggcccctccatcgtccaccgcaaatgcttctgaATCTGACCGAGTTCCCCGCGCCAGCGGGGATAAACCGCGCCAGCTTCGCGACGAGTTCCCCGCGCCAGCGGGGATAAACCGCGCCGGATCTGACCGAGTTCCCCGCGCCAGCGGGGATAAACCGCGCCAGCTTCGCGACGAGTTCCCCGCGCCAGCGGGGATAAACCGCGCCGGATCCATATGTAAGTAAGTAAGCGGCCGCGACTCTAGATCATAATCAGCCATACCACATTTGTAGAGGTTTTACTTGCTTTAAAAAACCTCCCACACCTCCCCCTGAACCTGAAACATAAAATGAATGCAATTGTTGTTGTT

**Supplementary Figure S13**

**Supplementary Figure S13.** Actin-8×CBS-GK construct.

The nucleotide sequences of *ACTB* and CBS are marked in orange and blue, respectively.

GGTTTAGTGAACCGTCAGATCCGCTAGCGCCACCATGgatgatgatatcgccgcgctcgtcgtcgacaacggctccggcatgtgcaaggccggcttcgcgggcgacgatgccccccgggccgtcttcccctccatcgtggggcgccccaggcaccagggcgtgatggtgggcatgggtcagaaggattcctatgtgggcgacgaggcccagagcaagagaggcatcctcaccctgaagtaccccatcgagcacggcatcgtcaccaactgggacgacatggagaaaatctggcaccacaccttctacaatgagctgcgtgtggctcccgaggagcaccccgtgctgctgaccgaggcccccctgaaccccaaggccaaccgcgagaagatgacccagatcatgtttgagaccttcaacaccccagccatgtacgttgctatccaggctgtgctatccctgtacgcctctggccgtaccactggcatcgtgatggactccggtgacggggtcacccacactgtgcccatctacgaggggtatgccctcccccatgccatcctgcgtctggacctggctggccgggacctgactgactacctcatgaagatcctcaccgagcgcggctacagcttcaccaccacggccgagcgggaaatcgtgcgtgacattaaggagaagctgtgctacgtcgccctggacttcgagcaagagatggccacggctgcttccagctcctccctggagaagagctacgagctgcctgacggccaggtcatcaccattggcaatgagcggttccgctgccctgaggcactcttccagccttccttcctgggcatggagtcctgtggcatccacgaaactaccttcaactccatcatgaagtgtgacgtggacatccgcaaagacctgtacgccaacacagtgctgtctggcggcaccaccatgtaccctggcattgccgacaggatgcagaaggagatcactgccctggcacccagcacaatgaagatcaagatcattgctcctcctgagcgcaagtactccgtgtggatcggcggctccatcctggcctcgctgtccaccttccagcagatgtggatcagcaagcaggagtatgacgagtccggcccctccatcgtccaccgcaaatgcttctgaGATCTGACCGAGTTCCCCGCGCCAGCGGGGATAAACCGCGCCAGCTTCGCGACGAGTTCCCCGCGCCAGCGGGGATAAACCGCGCCGGATCTGACCGAGTTCCCCGCGCCAGCGGGGATAAACCGCGCCAGCTTCGCGACGAGTTCCCCGCGCCAGCGGGGATAAACCGCGCCGGATCTGACCGAGTTCCCCGCGCCAGCGGGGATAAACCGCGCCAGCTTCGCGACGAGTTCCCCGCGCCAGCGGGGATAAACCGCGCCGGATCTGACCGAGTTCCCCGCGCCAGCGGGGATAAACCGCGCCAGCTTCGCGACGAGTTCCCCGCGCCAGCGGGGATAAACCGCGCCGGATCCATATGTAAGTAAGTAAGCGGCCGCGACTCTAGATCATAATCAGCCATACCACATTTGTAGAGGTTTTACTTGCTTTAAAAAACCTCCCACACCTCCCCCTGAACCTGAAACATAAAATGAATGCAATTGTTGTTGTT

**Supplementary Figure S14**

**Supplementary Figure S14.** Actin-16×CBS-GK construct.

The nucleotide sequences of *ACTB* and CBS are marked in orange and blue, respectively.

GGTTTAGTGAACCGTCAGATCCGCTAGCGCCACCATGgatgatgatatcgccgcgctcgtcgtcgacaacggctccggcatgtgcaaggccggcttcgcgggcgacgatgccccccgggccgtcttcccctccatcgtggggcgccccaggcaccagggcgtgatggtgggcatgggtcagaaggattcctatgtgggcgacgaggcccagagcaagagaggcatcctcaccctgaagtaccccatcgagcacggcatcgtcaccaactgggacgacatggagaaaatctggcaccacaccttctacaatgagctgcgtgtggctcccgaggagcaccccgtgctgctgaccgaggcccccctgaaccccaaggccaaccgcgagaagatgacccagatcatgtttgagaccttcaacaccccagccatgtacgttgctatccaggctgtgctatccctgtacgcctctggccgtaccactggcatcgtgatggactccggtgacggggtcacccacactgtgcccatctacgaggggtatgccctcccccatgccatcctgcgtctggacctggctggccgggacctgactgactacctcatgaagatcctcaccgagcgcggctacagcttcaccaccacggccgagcgggaaatcgtgcgtgacattaaggagaagctgtgctacgtcgccctggacttcgagcaagagatggccacggctgcttccagctcctccctggagaagagctacgagctgcctgacggccaggtcatcaccattggcaatgagcggttccgctgccctgaggcactcttccagccttccttcctgggcatggagtcctgtggcatccacgaaactaccttcaactccatcatgaagtgtgacgtggacatccgcaaagacctgtacgccaacacagtgctgtctggcggcaccaccatgtaccctggcattgccgacaggatgcagaaggagatcactgccctggcacccagcacaatgaagatcaagatcattgctcctcctgagcgcaagtactccgtgtggatcggcggctccatcctggcctcgctgtccaccttccagcagatgtggatcagcaagcaggagtatgacgagtccggcccctccatcgtccaccgcaaatgcttctgaGATCTCGACCGAGTTCCCCGCGCCAGCGGGGATAAACCGCGCCAGCTTCGAATTCTGCAGTCGAAGAGTTCCCCGCGCCAGCGGGGATAAACCCGATCCACCGGTATTTATCTCGACCGAGTTCCCCGCGCCAGCGGGGATAAACCGCGCCAGCTTCGAATTCGAGTTCCCCGCGCCAGCGGGGATAAACCCGATCCACCGGTATTTATCTCGACCGAGTTCCCCGCGCCAGCGGGGATAAACCGCGCCAGCTTCGAATTCTGCAGTCGAAGAGTTCCCCGCGCCAGCGGGGATAAACCCGATCCACCGGTATTTATCTCGACCGAGTTCCCCGCGCCAGCGGGGATAAACCGCGCCAGCTTCGAATTCGAGTTCCCCGCGCCAGCGGGGATAAACCCGATCCACCGGTATTTATCTCGACCGAGTTCCCCGCGCCAGCGGGGATAAACCGCGCCAGCTTCGAATTCTGCAGTCGAAGAGTTCCCCGCGCCAGCGGGGATAAACCCGATCCACCGGTATTTATCTCGACCGAGTTCCCCGCGCCAGCGGGGATAAACCGCGCCAGCTTCGAATTCGAGTTCCCCGCGCCAGCGGGGATAAACCCGATCCACCGGTATTTATCTCGACCGAGTTCCCCGCGCCAGCGGGGATAAACCGCGCCAGCTTCGAATTCTGCAGTCGAAGAGTTCCCCGCGCCAGCGGGGATAAACCCGATCCACCGGTATTTATCTCGACCGAGTTCCCCGCGCCAGCGGGGATAAACCGCGCCAGCTTCGAATTCGAGTTCCCCGCGCCAGCGGGGATAAACCCGATCCACCGGTATTTATCTCGAGCTCAAGCTTCGAATTCTGCAGTCGACGGTACCGCGGGCCCGGGATCCATATGTAAGTAAGTAAGCGGCCGCGACTCTAGATCATAATCAGCCATACCACATTTGTAGAGGTTTTACTTGCTTTAAAAAACCTCCCACACCTCCCCCTGAACCTGAAACATAAAATGAATGCAATTGTTGTTGTT

**Supplementary Figure S15**

**Supplementary Figure S15.** hTERC-16×CBS-GK construct.

The nucleotide sequences of *hTERC* and CBS are marked in green and blue, respectively.

GGTTTAGTGAACCGTCAGATCCGCTAGCTTTATAAGCCGACTCGCCCGGCAGCGCACCgggttgcggagggtgggcctgggaggggtggtggccattttttgtctaaccctaactgagaagggcgtaggcgccgtgcttttgctccccgcgcgctgtttttctcgctgactttcagcgggcggaaaagcctcggcctgccgccttccaccgttcattctagagcaaacaaaaaatgtcagctgctggcccgttcgcccctcccggggacctgcggcgggtcgcctgcccagcccccgaaccccgcctggaggccgcggtcggcccggggcttctccggaggcacccactgccaccgcgaagagttgggctctgtcagccgcgggtctctcgggggcgagggcgaggttcaggcctttcaggccgcaggaagaggaacggagcgagtccccgcgcgcggcgcgattccctgagctgtgggacgtgcacccaggactcggctcacacatgcAGATCTCGACCGAGTTCCCCGCGCCAGCGGGGATAAACCGCGCCAGCTTCGAATTCTGCAGTCGAAGAGTTCCCCGCGCCAGCGGGGATAAACCCGATCCACCGGTATTTATCTCGACCGAGTTCCCCGCGCCAGCGGGGATAAACCGCGCCAGCTTCGAATTCGAGTTCCCCGCGCCAGCGGGGATAAACCCGATCCACCGGTATTTATCTCGACCGAGTTCCCCGCGCCAGCGGGGATAAACCGCGCCAGCTTCGAATTCTGCAGTCGAAGAGTTCCCCGCGCCAGCGGGGATAAACCCGATCCACCGGTATTTATCTCGACCGAGTTCCCCGCGCCAGCGGGGATAAACCGCGCCAGCTTCGAATTCGAGTTCCCCGCGCCAGCGGGGATAAACCCGATCCACCGGTATTTATCTCGACCGAGTTCCCCGCGCCAGCGGGGATAAACCGCGCCAGCTTCGAATTCTGCAGTCGAAGAGTTCCCCGCGCCAGCGGGGATAAACCCGATCCACCGGTATTTATCTCGACCGAGTTCCCCGCGCCAGCGGGGATAAACCGCGCCAGCTTCGAATTCGAGTTCCCCGCGCCAGCGGGGATAAACCCGATCCACCGGTATTTATCTCGACCGAGTTCCCCGCGCCAGCGGGGATAAACCGCGCCAGCTTCGAATTCTGCAGTCGAAGAGTTCCCCGCGCCAGCGGGGATAAACCCGATCCACCGGTATTTATCTCGACCGAGTTCCCCGCGCCAGCGGGGATAAACCGCGCCAGCTTCGAATTCGAGTTCCCCGCGCCAGCGGGGATAAACCCGATCCACCGGTATTTATCTCGAGCTCAAGCTTCGAATTCTGCAGTCGACGGTACCGCGGGCCCGGGATCCATATGTAAGTAAGTAAGCGGCCGCGACTCTAGATCATAATCAGCCATACCACATTTGTAGAGGTTTTACTTGCTTTAAAAAACCTCCCACACCTCCCCCTGAACCTGAAACATAAAATGAATGCAATTGTTGTTGTT

**Supplementary Figure S16**

**Supplementary Figure S16.** U6-1×CBS construct.

The nucleotide sequence of U6 promoter and CBS are marked in red and blue, respectively.

GAGGGCCTATTTCCCATGATTCCTTCATATTTGCATATACGATACAAGGCTGTTAGAGAGATAATTGGAATTAATTTGACTGTAAACACAAAGATATTAGTACAAAATACGTGACGTAGAAAGTAATAATTTCTTGGGTAGTTTGCAGTTTTAAAATTATGTTTTAAAATGGACTATCATATGCTTACCGTAACTTGAAAGTATTTCGATTTCTTGGCTTTATATATCTTGTGGAAAGGACGAAACACCGGAGTTCCCCGCGCCAGCGGGGATAAACCGAAACTGACGGGCTAATGCTTGCATTTTTTT

**Supplementary Figure S17**

**Supplementary Figure S17.** GK construct.

The GK plasmid is used as backbone plasmid in this study, which is obtained by deleting EGFP CDS in pEGFP-N1. The nucleotide sequence of multiple cloning site (MCS) is marked in blue.

GGTTTAGTGAACCGTCAGATCCGCTAGCGCTACCGGACTCAGATCTCGAGCTCAAGCTTCGAATTCTGCAGTCGACGGTACCGCGGGCCCGGGATCCATATGTAAGTAAGTAAGCGGCCGCGACTCTAGATCATAATCAGCCATACCACATTTGTAGAGGTTTTACTTGCTTTAAAAAACCTCCCACACCTCCCCCTGAACCTGAAACATAAAATGAATGCAATTGTTGTTGTT

**Supplementary Figure S18**

**Supplementary Figure S18.** Rm-4×CBS variants series constructs.

The nucleotide sequences of *DsRed-monomer* (abbreviated as *Rm*) gene and CBS variants are marked in red and blue, respectively.

ctctggctaactagagaacccactgcttactggcttaCTAGCTAGCACCGGTCGCCACCATGgacaacaccgaggacgtcatcaaggagttcatgcagttcaaggtgcgcatggagggctccgtgaacggccactacttcgagatcgagggcgagggcgagggcaagccctacgagggcacccagaccgccaagctgcaggtgaccaagggcggccccctgcccttcgcctgggacatcctgtccccccagttccagtacggctccaaggcctacgtgaagcaccccgccgacatccccgactacatgaagctgtccttccccgagggcttcacctgggagcgctccatgaacttcgaggacggcggcgtggtggaggtgcagcaggactcctccctgcaggacggcaccttcatctacaaggtgaagttcaagggcgtgaacttccccgccgacggccccgtaatgcagaagaagactgccggctgggagccctccaccgagaagctgtacccccaggacggcgtgctgaagggcgagatctcccacgccctgaagctgaaggacggcggccactacacctgcgacttcaagaccgtgtacaaggccaagaagcccgtgcagctgcccggcaaccactacgtggactccaagctggacatcaccaaccacaacgaggactacaccgtggtggagcagtacgagcacgccgaggcccgccactccggctcccagtccggactcagatctcgactttgaGCGCTACCGGACTCAGATCTCGACCAGGAGTGGAAAGGACGAAACACCGNNNNNNNNNNNNNNNNNNNNNNNNNNNNNTTTTTTTGGCACTTTTCGGGGAAATGTGCGCGGAACCCCTATTTGTTTGCAAGTGGAAAGGACGAAACACCGNNNNNNNNNNNNNNNNNNNNNNNNNNNNNTTTTTTTGGCACTTTTCGGGGAAATGTGCGCGGAACCCCTATTTGTTTACTAGTGGAAAGGACGAAACACCGNNNNNNNNNNNNNNNNNNNNNNNNNNNNNTTTTTTTGGCACTTTTCGGGGAAATGTGCGCGGAACCCCTATTTGTTTCAGAGTGGAAAGGACGAAACACCGNNNNNNNNNNNNNNNNNNNNNNNNNNNNNTTTTTTTGGCACTTTTCGGGGAAATGTGCGCGGAACCCCTATTTGTTTTGTGGCTCGAGTCTAGAGGGCCCGTTTAAACCCGCTGATCAGCCTCGA

The Rm-4×CBS variants series plasmids include 24 plasmids, which share the same vector backbone and differ on CBS sequences. The plasmid ID and the corresponding CBS sequences are as follows:

**Plasmid ID: CBS variants sequence (5’-3’)**

Rm-4×*Ec*CBS: GAGTTCCCCGCGCCAGCGGGGATAAACCG

Rm-4×*Tt*CBS: GTAGTCCCCACGCGTGTGGGGATGGACCG

Rm-4×*Sa*CBS: GTCCTCCCCACGCACGTGGGGGTGCTCCG

Rm-4×*Ct*CBS: GTCTTCCCCACGCCCGTGGGGGTGTTTC

Rm-4×*Cd*CBS: GTCTTCTCCGCACACGCGGAGGTATTTC

Rm-4×*Ec*CBS-U5A: GAGTACCCCGCGCCAGCGGGGATAAACCG

Rm-4×*Ec*CBS-A22U: GAGTTCCCCGCGCCAGCGGGGTTAAACCG

Rm-4×*Ec*CBS-U23A: GAGTTCCCCGCGCCAGCGGGGAAAAACCG

Rm-4×*Ec*CBS-A24U: GAGTTCCCCGCGCCAGCGGGGATTAACCG

Rm-4×*Ec*CBS-C14G: GAGTTCCCCGCGCGAGCGGGGATAAACCG

Rm-4×*Ec*CBS-A15U: GAGTTCCCCGCGCCTGCGGGGATAAACCG

Rm-4×*Ec*CBS-C14G|A15U: GAGTTCCCCGCGCGTGCGGGGATAAACCG

Rm-4×*Ec*CBS-G16C: GAGTTCCCCGGGCCAGCGGGGATAAACCG

Rm-4×*Ec*CBS-G19C: GAGTTCCGCGCGCCAGCGGGGATAAACCG

Rm-4×*Ec*CBS-G21C: GAGTTGCCCGCGCCAGCGGGGATAAACCG

Rm-4×*Ec*CBS-stem-mut1: GAGTTGGGGCGGCCACGCCCCATAAACCG

Rm-4×*Ec*CBS-stem-mut2: GAGTTAAAATAGCCATATTTTATAAACCG

Rm-4×*Ec*CBS-stem_-1: GAGTTCCCGCGCCAGCGGGATAAACCG

Rm-4×*Ec*CBS-stem_-2: GAGTTCCGCGCCAGCGGATAAACCG

Rm-4×*Ec*CBS-stem_+1 : GAGTTCCCCCGCGCCAGCGGGGGATAAACCG

Rm-4×*Ec*CBS-stem_+2 : GAGTTCCCCCCGCGCCAGCGGGGGGATAAACCG

Rm-4×*Ec*CBS-G12C: GAGTTCCCCGCCCCAGCGGGGATAAACCG

Rm-4×*Ec*CBS-C13G: GAGTTCCCCGCGGCAGCGGGGATAAACCG

Rm-4×*Ec*CBS-G12C|C13G: GAGTTCCCCGCCGCAGCGGGGATAAACCG

**Supplementary Figure S19**


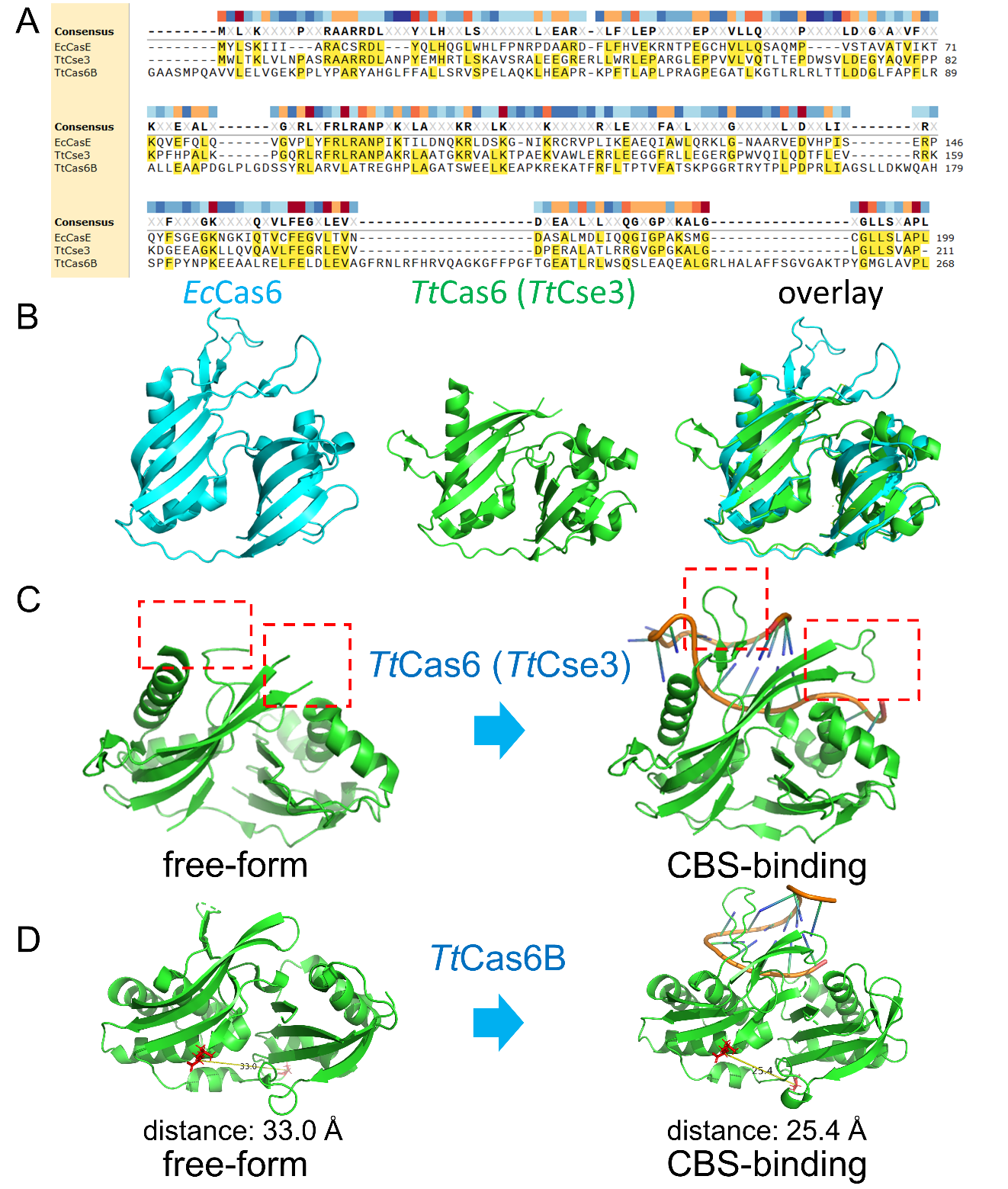


**Supplementary Figure S19. CBS-binding induces an allosteric change of Cas6.** (**A**) Alignments of *Ec*Cas6, *Tt*Cas6 and *Tt*Cas6B amino acid sequences. (**B**) Structural comparison of free-form *Ec*Cas6 and *Tt*Cas6. (**C**) CBS binding facilitates *Tt*Cas6 stability. Two loops connecting β-strands (marked with red box) are stabilized after binding CBS. Free-form *Tt*Cas6 PDB: 1wj9; CBS-binding *Tt*Cas6 PDB: 2y8w. (**D**) Distance of *Tt*Cas6B’s N and C ends changes after binding CBS. The N terminal and C terminal amino acids were marked with red and shown in stick form. *Ec* for *Escherichia coli*; Tt for *Thermus thermophilus*. The distance was measured with PyMOL software. Free-form *Tt*Cas6B PDB: 4c98; CBS-binding *Tt*Cas6B PDB: 4c9d.

**Supplementary Figure S20**

**
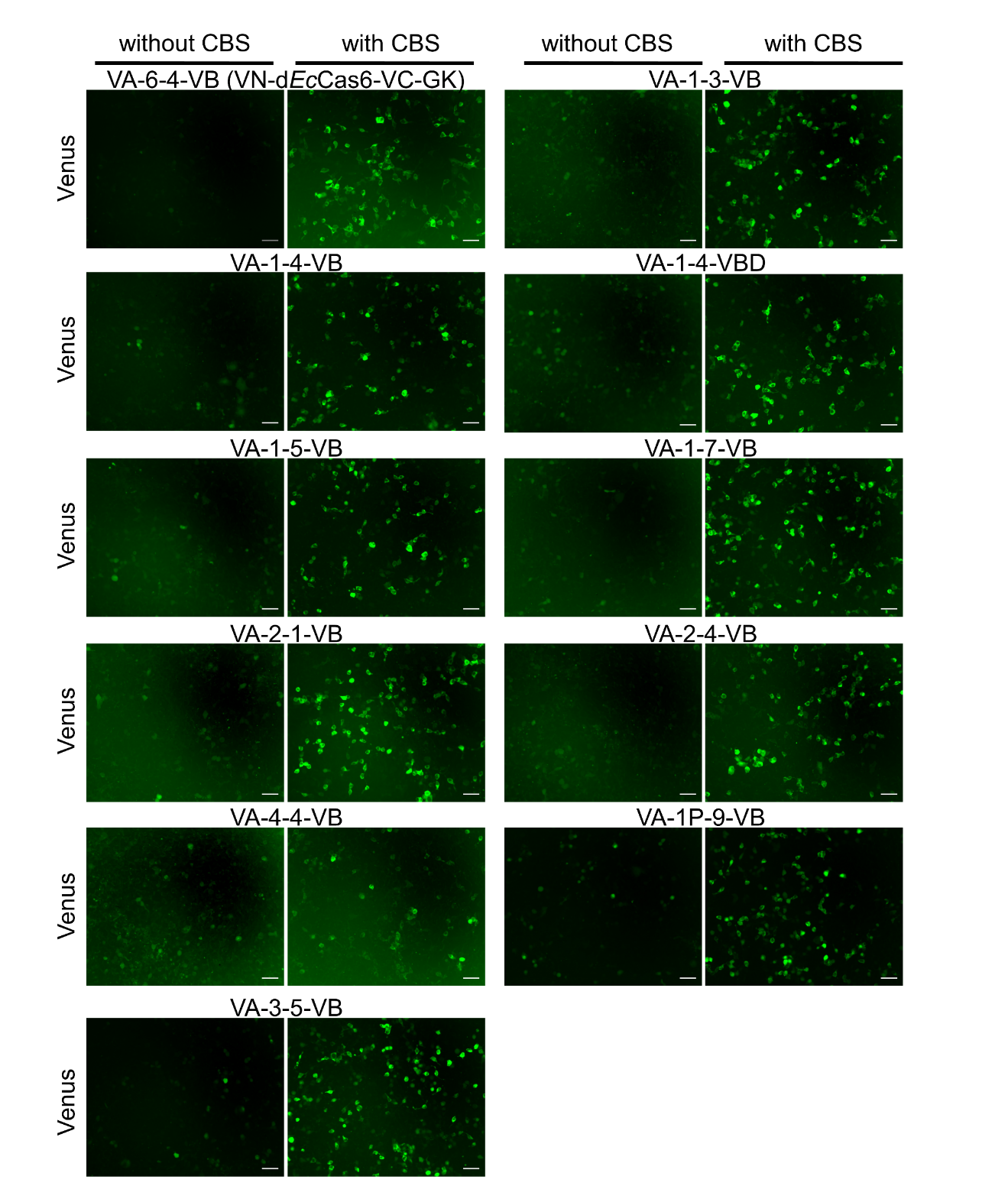
**

**Supplementary Figure S20. Tests of diverse linkers on Cas6FC RNA tracking.** Without CBS groups are co-transfection of pDsRed-Monomer-C1 and VA-x-x-VB-GK series plasmids; With CBS groups are co-transfection of Rm- 20×CBS-C1 and VA-x-x-VB-GK series plasmids. Plasmid dosages are:100 ng VA-x-x-VB-GK and 400 ng pDsRed-Monomer-C1 or Rm- 20×CBS-C1. Scale bar, 50 μm

The amino acid sequences of VA-x-x-VB series plasmids are listed as follows:

**VA (N terminal fragment of Venus: 1-153 aa):** MVSKGEELFTGVVPILVELDGDVNGHKFSVSGEGEGDATYGKLTLKLICTTGKLPVPWPTLVTTLGYGLQCFARYPDHMKQHDFFKSAMPEGYVQERTIFFKDDGNYKTRAEVKFEGDTLVNRIELKGIDFKEDGNILGHKLEYNYNSHNVYIT

**VB (C terminal fragment of Venus: 153-238 aa):** ADKQKNGIKANFKIRHNIEDGSVQLADHYQQNTPIGDGPVLLPDNHYLSYQSALSKDPNEKRDHMVLLEFVTAAGITLGMDELYK

**VBD** (**C terminal fragment of Venus: 153-238 aa, with mutation G175S**): ADKQKNGIKANFKIRHNIEDGSVQLADHYQQNTPIGDGPVLLPDNHYLSYQSALSKDPNEKRDHMVLLEFVTAAGITLGMDELYK

**linker1**: SSGSSGSGSKLAT

**linker1P**: RSEFGGGGSPGGGGSPGGGGSKLAT

**linker2**: LEAEAAAKEAAAKEAAAKAKLAT

**linker3**: LQSTPAPAPAPAP

**linker4**: LQSTTCTAGAEF

**linker5**: LQSTVPRSS

**linker6**: LERPACKIPNDLKQKVMNHKLAT

**linker7**: LQSTGGGAATTCN

**linker9**: LQSTVPRARDP

**Supplementary Figure S21**

**
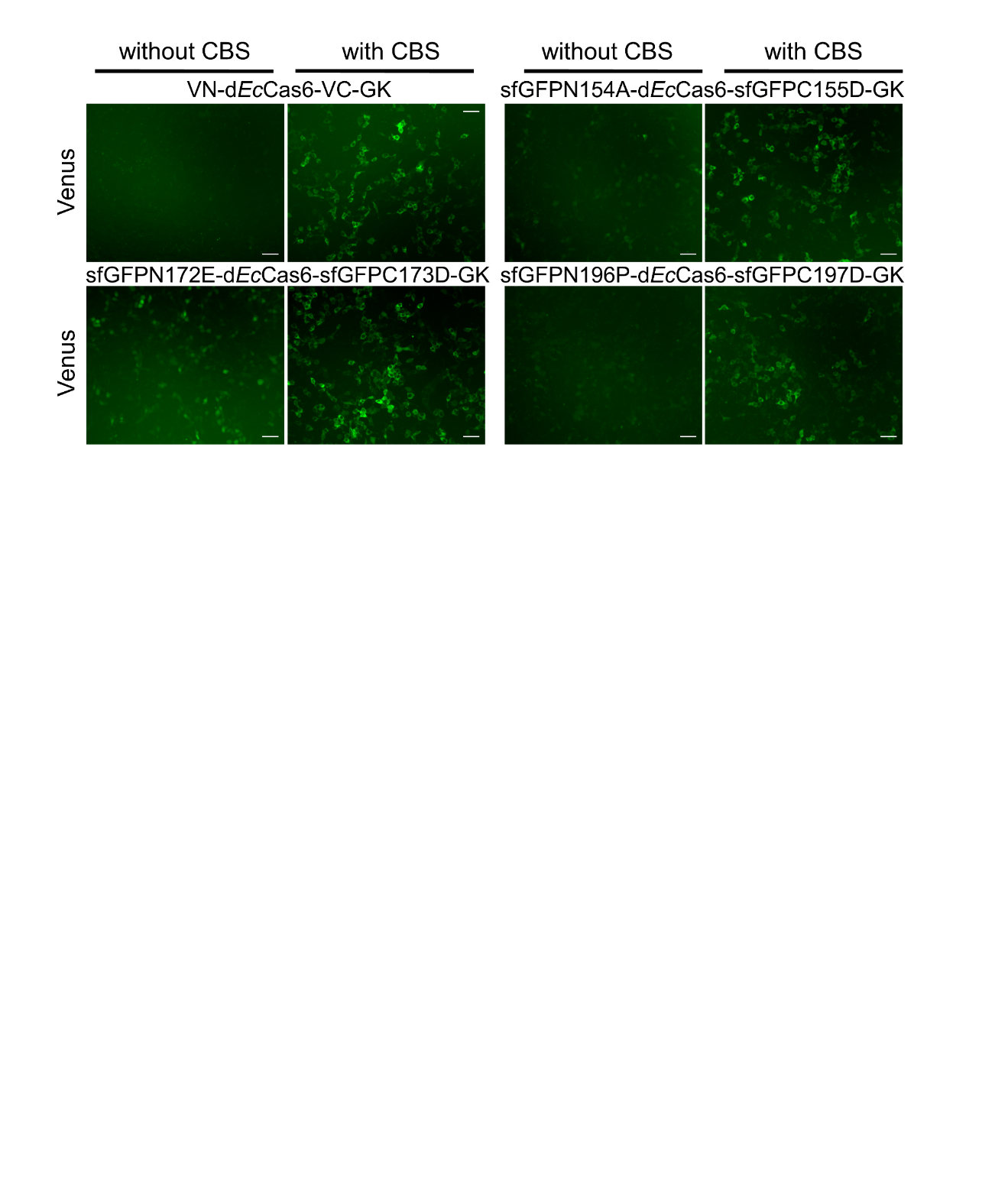
**

**Supplementary Figure S21. sfGFP-based Cas6FC vs Venus-based Cas6FC RNA tracking.** Without CBS groups are co-transfection of pDsRed-Monomer-C1 and VN-d*Ec*Cas6-VC-GK or sfGFPNx-dEcCas6-sfGFPCx-GK series plasmids; With CBS groups are co-transfection of Rm- 20×CBS-C1 and sfGFPNx-dEcCas6-sfGFPCx-GK series plasmids. Plasmid dosages are:100 ng VN-d*Ec*Cas6-VC-GK or sfGFPNx-dEcCas6-sfGFPCx-GK series plasmids and 400 ng pDsRed-Monomer-C1 or Rm- 20×CBS-C1. Scale bar, 50 μm

The amino acid sequences of sfGFPNx-dEcCas6-sfGFPCx-GK series plasmids are listed as follows:

**sfGFPN154A (N terminal fragment of sfGFP: 1-154 aa):** MSKGEELFTGVVPILVELDGDVNGHKFSVRGEGEGDATNGKLTLKFICTTGKLPVPWPTLVTTLTYGVQCFSRYPDHMKRHDFFKSAMPEGYVQERTISFKDDGTYKTRAEVKFEGDTLVNRIELKGIDFKEDGNILGHKLEYNFNSHNVYITA

**sfGFPC155D (C terminal fragment of sfGFP: 155-238 aa):** DKQKNGIKANFKIRHNVEDGSVQLADHYQQNTPIGDGPVLLPDNHYLSTQSVLSKDPNEKRDHMVLLEFVTAAGITHGMDELYK

**sfGFPN172E (N terminal fragment of sfGFP: 1-172 aa):** MSKGEELFTGVVPILVELDGDVNGHKFSVRGEGEGDATNGKLTLKFICTTGKLPVPWPTLVTTLTYGVQCFSRYPDHMKRHDFFKSAMPEGYVQERTISFKDDGTYKTRAEVKFEGDTLVNRIELKGIDFKEDGNILGHKLEYNFNSHNVYITADKQKNGIKANFKIRHNVE

**sfGFPC173D (C terminal fragment of sfGFP: 173-238 aa):** DGSVQLADHYQQNTPIGDGPVLLPDNHYLSTQSVLSKDPNEKRDHMVLLEFVTAAGITHGMDELYK

**sfGFPN196P (N terminal fragment of sfGFP: 1-196 aa):** MSKGEELFTGVVPILVELDGDVNGHKFSVRGEGEGDATNGKLTLKFICTTGKLPVPWPTLVTTLTYGVQCFSRYPDHMKRHDFFKSAMPEGYVQERTISFKDDGTYKTRAEVKFEGDTLVNRIELKGIDFKEDGNILGHKLEYNFNSHNVYITADKQKNGIKANFKIRHNVEDGSVQLADHYQQNTPIGDGPVLLP

**sfGFPC197D (C terminal fragment of sfGFP: 197-238 aa):** DNHYLSTQSVLSKDPNEKRDHMVLLEFVTAAGITHGMDELYK

**Supplementary Figure S22**

**
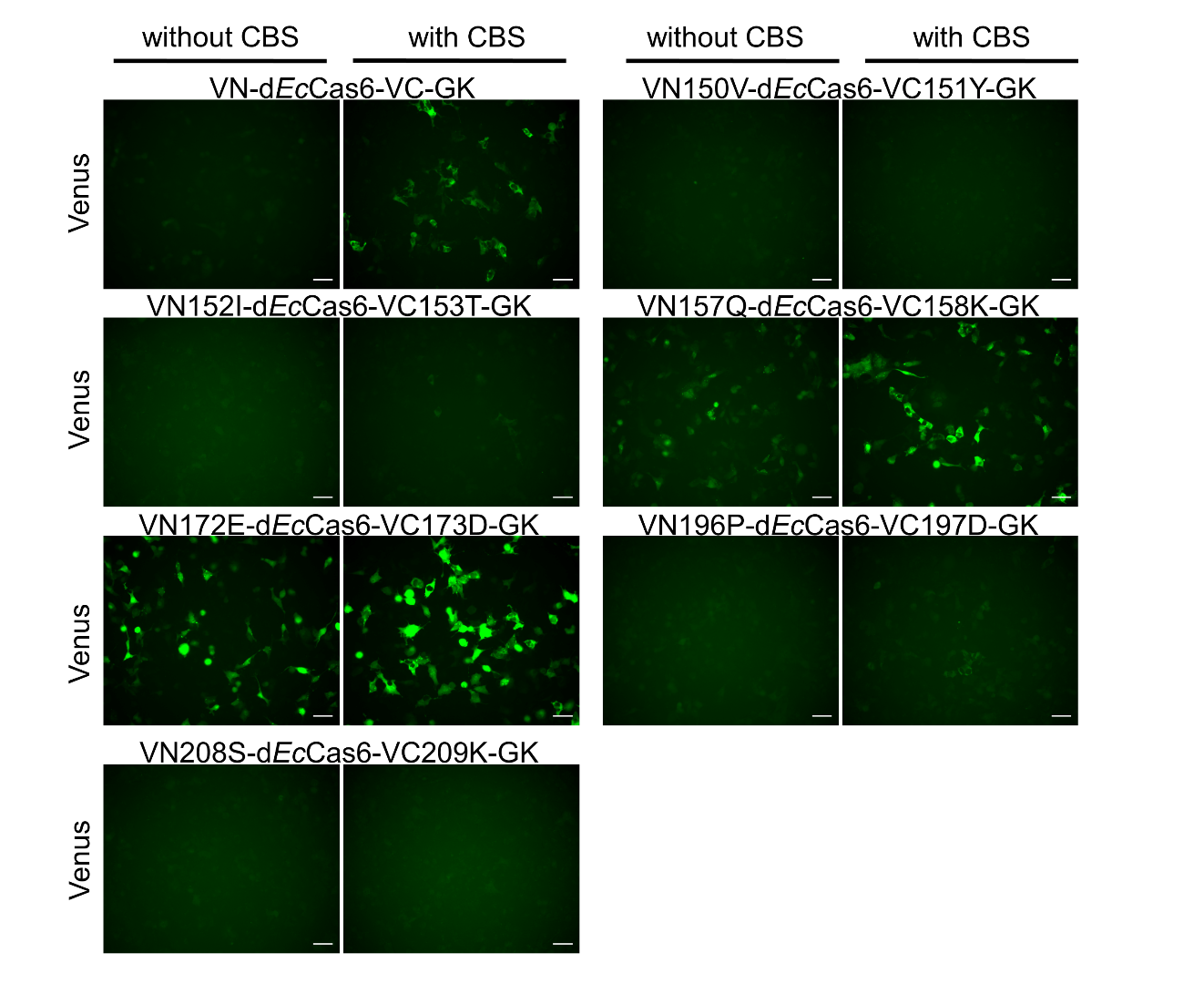
**

**Supplementary Figure S22.** **Tests of diverse split-complementation strategies of venus for Cas6FC.** Without CBS groups are co-transfection of pDsRed-Monomer-C1 and VNx-d*Ec*Cas6-VCx-GK series plasmids; With CBS groups are co-transfection of Rm- 20×CBS-C1 and VNx-d*Ec*Cas6-VCx-GK series plasmids. Plasmid dosages are:100 ng VNx-d*Ec*Cas6-VCx-GK series plasmids and 400 ng pDsRed-Monomer-C1 or Rm- 20×CBS-C1. Scale bar, 50 μm

The amino acid sequences of VNx-d*Ec*Cas6-VCx-GK series plasmids are listed as follows:

**VN150V (N terminal fragment of sfGFP: 1-150 aa):** MVSKGEELFTGVVPILVELDGDVNGHKFSVSGEGEGDATYGKLTLKLICTTGKLPVPWPTLVTTLGYGLQCFARYPDHMKQHDFFKSAMPEGYVQERTIFFKDDGNYKTRAEVKFEGDTLVNRIELKGIDFKEDGNILGHKLEYNYNSHNV

**VC151Y (C terminal fragment of sfGFP: 151-238 aa):** YITADKQKNGIKANFKIRHNIEDGGVQLADHYQQNTPIGDGPVLLPDNHYLSYQSALSKDPNEKRDHMVLLEFVTAAGITLGMDELYK

**VN152I (N terminal fragment of sfGFP: 1-152 aa):** MVSKGEELFTGVVPILVELDGDVNGHKFSVSGEGEGDATYGKLTLKLICTTGKLPVPWPTLVTTLGYGLQCFARYPDHMKQHDFFKSAMPEGYVQERTIFFKDDGNYKTRAEVKFEGDTLVNRIELKGIDFKEDGNILGHKLEYNYNSHNVYI

**VC153T (C terminal fragment of sfGFP: 153-238 aa):** TADKQKNGIKANFKIRHNIEDGGVQLADHYQQNTPIGDGPVLLPDNHYLSYQSALSKDPNEKRDHMVLLEFVTAAGITLGMDELYK

**VN157Q (N terminal fragment of sfGFP: 1-157 aa):** MVSKGEELFTGVVPILVELDGDVNGHKFSVSGEGEGDATYGKLTLKLICTTGKLPVPWPTLVTTLGYGLQCFARYPDHMKQHDFFKSAMPEGYVQERTIFFKDDGNYKTRAEVKFEGDTLVNRIELKGIDFKEDGNILGHKLEYNYNSHNVYITADKQ

**VC158K (C terminal fragment of sfGFP: 158-238 aa):** KNGIKANFKIRHNIEDGGVQLADHYQQNTPIGDGPVLLPDNHYLSYQSALSKDPNEKRDHMVLLEFVTAAGITLGMDELYK

**VN172E (N terminal fragment of sfGFP: 1-196 aa):** MVSKGEELFTGVVPILVELDGDVNGHKFSVSGEGEGDATYGKLTLKLICTTGKLPVPWPTLVTTLGYGLQCFARYPDHMKQHDFFKSAMPEGYVQERTIFFKDDGNYKTRAEVKFEGDTLVNRIELKGIDFKEDGNILGHKLEYNYNSHNVYITADKQKNGIKANFKIRHNIE

**VC173D (C terminal fragment of sfGFP: 197-238 aa):** DGGVQLADHYQQNTPIGDGPVLLPDNHYLSYQSALSKDPNEKRDHMVLLEFVTAAGITLGMDELYK

**VN196P (N terminal fragment of sfGFP: 1-196 aa):** MVSKGEELFTGVVPILVELDGDVNGHKFSVSGEGEGDATYGKLTLKLICTTGKLPVPWPTLVTTLGYGLQCFARYPDHMKQHDFFKSAMPEGYVQERTIFFKDDGNYKTRAEVKFEGDTLVNRIELKGIDFKEDGNILGHKLEYNYNSHNVYITADKQKNGIKANFKIRHNIEDGGVQLADHYQQNTPIGDGPVLLP

**VC197D (C terminal fragment of sfGFP: 197-238 aa):** DNHYLSYQSALSKDPNEKRDHMVLLEFVTAAGITLGMDELYK

**VN208S (N terminal fragment of sfGFP: 1-196 aa):** MVSKGEELFTGVVPILVELDGDVNGHKFSVSGEGEGDATYGKLTLKLICTTGKLPVPWPTLVTTLGYGLQCFARYPDHMKQHDFFKSAMPEGYVQERTIFFKDDGNYKTRAEVKFEGDTLVNRIELKGIDFKEDGNILGHKLEYNYNSHNVYITADKQKNGIKANFKIRHNIEDGGVQLADHYQQNTPIGDGPVLLPDNHYLSYQSALS

**VC209K (C terminal fragment of sfGFP: 197-238 aa):** KDPNEKRDHMVLLEFVTAAGITLGMDELYK

**Supplementary Figure S23**


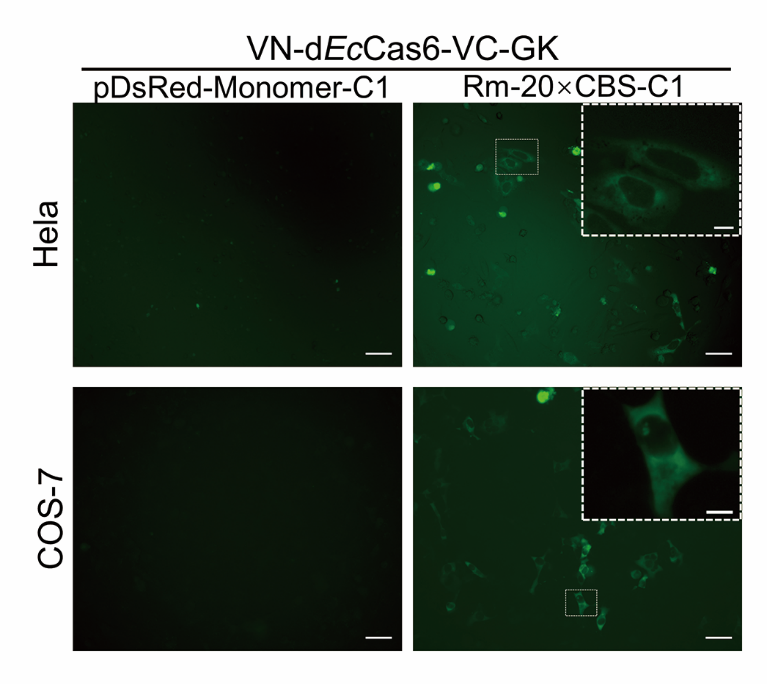


**Supplementary Figure S23.** Applications of Cas6FC RNA tracking system in Hela and COS-7 cell lines. Cas6FC system tracked CBS tagged *Rm (DsRed-Monomer)* mRNA in Hela and COS-7 cell lines. The plasmids dosages are: 100 ng VN-d*Ec*Cas6-VC-GK and 400 ng pDsRed-Monomer-C1 or Rm-20×CBS-C1. The images were captured with ZEISS Vert.A1 microscope. Scale bar in large field, 50 μm; scale bar in small field, 10 μm.

**Supplementary Figure S24**


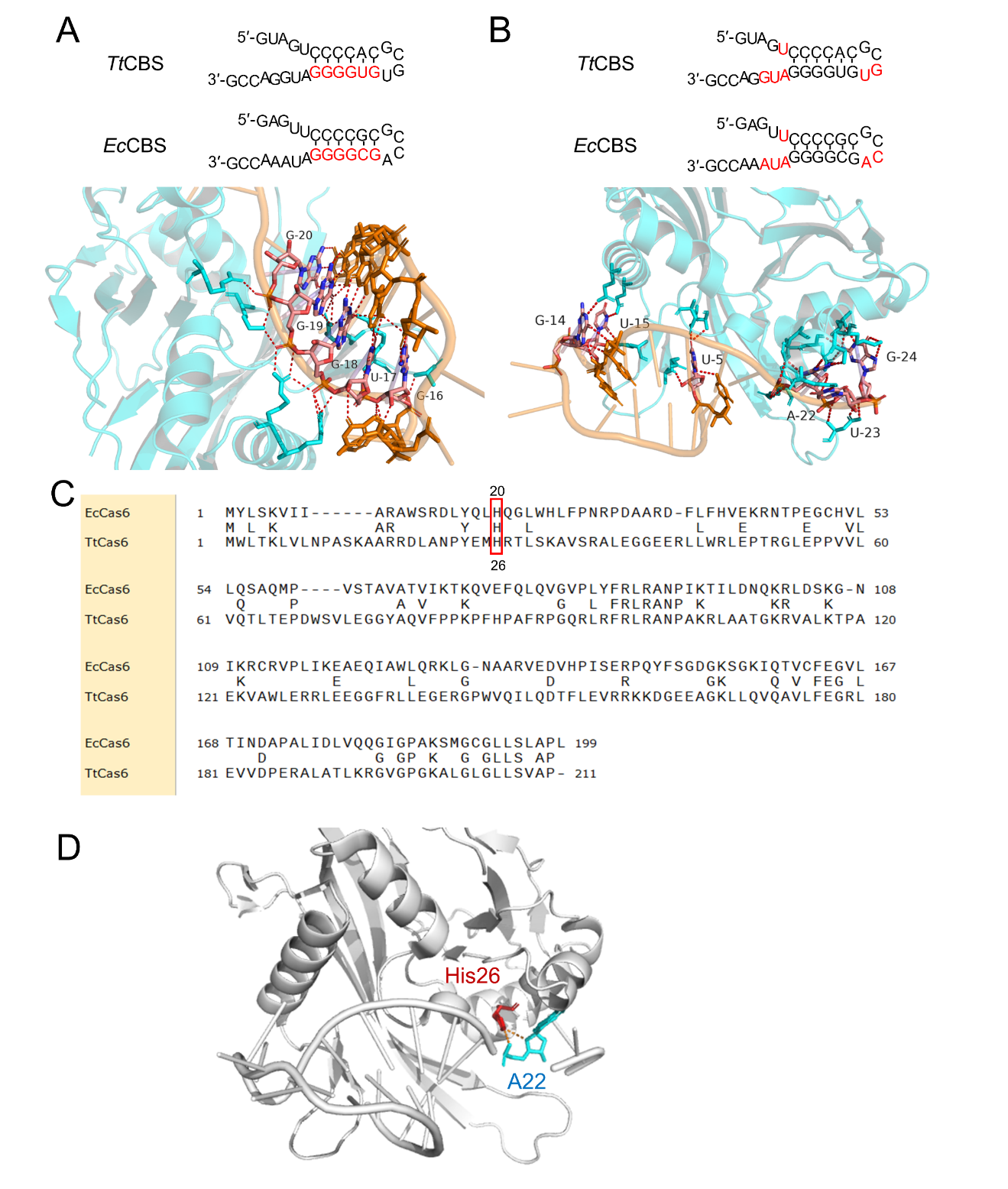


**Supplementary Figure S24.** The nucleotides of CBS interacting with Cas6. (**A**) The top panel shows *Tt*CBS’s non-stem nucleotides interacting with *Tt*Cas6, and *Ec*CBS’s corresponding putative nucleotides interacting with *Ec*Cas6. The bottom panel shows the phosphate backbones of nucleotides G16, U17, G18, G19, G20, and G21 interact with *Tt*Cas6 with hydrogen bonds. (**B**) The top panel shows *Tt*CBS’s non-stem nucleotides interacting with *Tt*Cas6, and *Ec*CBS’s corresponding putative nucleotides interacting with *Ec*Cas6. The bottom panel shows the bases of nucleotides U5, G14, U15, A22, U23 and G24 interact with *Tt*Cas6 with hydrogen bonds. (**C**) The alignment of *Ec*Cas6 and *Tt*Cas6 amino acid sequences. His26 in *Tt*Cas6 and the corresponding His20 in *Ec*Cas6 are marked with red box. (**D**) The interaction between His26 (marked with red) and A22 (marked with cyan) in *Tt*Cas6-*Tt*CBS structure. The above CBS-Cas6 interaction snapshots originate from *Tt*Cas6*-Tt*CBS cocrystal structure (PDB: 2y8w).
